# Insulator-donor electron wavefunction coupling in pseudo-bilayer organic solar cells achieving a certificated efficiency of 19.18%

**DOI:** 10.1093/nsr/nwae385

**Published:** 2024-10-30

**Authors:** Jiangkai Sun, Ruijie Ma, Xue Yang, Xiaoyu Xie, Dongcheng Jiang, Yuan Meng, Yiyun Li, Fengzhe Cui, Mengfei Xiao, Kangning Zhang, Yu Chen, Xinxin Xia, Maojie Zhang, Xiaoyan Du, Long Ye, Haibo Ma, Kun Gao, Feng Chen, Gang Li, Xiaotao Hao, Hang Yin

**Affiliations:** School of Physics, State Key Laboratory of Crystal Materials, Shandong University, Jinan 250100, China; Department of Electric and Electronic Engineering, Research Institute for Smart Energy (RISE), Photonic Research Institute (PRI), The Hong Kong Polytechnic University, Hong Kong 999077, China; School of Physics, State Key Laboratory of Crystal Materials, Shandong University, Jinan 250100, China; Qingdao Institute for Theoretical and Computational Sciences, School of Chemistry and Chemical Engineering, Shandong University, Qingdao 266237, China; School of Physics, State Key Laboratory of Crystal Materials, Shandong University, Jinan 250100, China; School of Physics, State Key Laboratory of Crystal Materials, Shandong University, Jinan 250100, China; School of Physics, State Key Laboratory of Crystal Materials, Shandong University, Jinan 250100, China; School of Physics, State Key Laboratory of Crystal Materials, Shandong University, Jinan 250100, China; School of Physics, State Key Laboratory of Crystal Materials, Shandong University, Jinan 250100, China; School of Physics, State Key Laboratory of Crystal Materials, Shandong University, Jinan 250100, China; Beijing Synchrotron Radiation Facility, Institute of High Energy Physics, Chinese Academy of Sciences, Beijing 100049, China; National Engineering Research Center for Colloidal Materials, School of Chemistry and Chemical Engineering, Shandong University, Jinan 250100, China; National Engineering Research Center for Colloidal Materials, School of Chemistry and Chemical Engineering, Shandong University, Jinan 250100, China; School of Physics, State Key Laboratory of Crystal Materials, Shandong University, Jinan 250100, China; School of Materials Science and Engineering, Tianjin Key Laboratory of Molecular Optoelectronic Sciences, Collaborative Innovation Center of Chemical Science and Engineering (Tianjin), Tianjin University, Tianjin 300350, China; Qingdao Institute for Theoretical and Computational Sciences, School of Chemistry and Chemical Engineering, Shandong University, Qingdao 266237, China; School of Physics, State Key Laboratory of Crystal Materials, Shandong University, Jinan 250100, China; School of Physics, State Key Laboratory of Crystal Materials, Shandong University, Jinan 250100, China; Department of Electric and Electronic Engineering, Research Institute for Smart Energy (RISE), Photonic Research Institute (PRI), The Hong Kong Polytechnic University, Hong Kong 999077, China; School of Physics, State Key Laboratory of Crystal Materials, Shandong University, Jinan 250100, China; School of Physics, State Key Laboratory of Crystal Materials, Shandong University, Jinan 250100, China

**Keywords:** organic semiconductors, polymeric insulators, electron transport, organic solar cells

## Abstract

The incorporation of polymeric insulators has led to notable achievements in the field of organic semiconductors. By altering the blending concentration, polymeric insulators exhibit extensive capabilities in regulating molecular configuration, film crystallinity, and mitigation of defect states. However, current research suggests that the improvement in such physical properties is primarily attributed to the enhancement of thin film morphology, an outcome that seems to be an inevitable consequence of incorporating insulators. Herein, we report a general and completely new effect of polymeric insulators in organic semiconductors: the insulator-donor electron wavefunction coupling effect. Such insulators can couple with donor polymers to reduce the energy barrier level and facilitate intramolecular electron transport. Besides the morphological effects, we observed that this coupling effect is another mechanism that can significantly enhance electron mobility (up to 100 times) through the incorporation of polymeric insulators in a series of donor systems. With this effect, we proposed a polymeric insulator blending approach to fabricate state-of-the-art pseudo-bilayer organic solar cells, and the PM6/L8-BO device exhibits a high efficiency of 19.50% (certificated 19.18%) with an improved interfacial electron transport property. This work not only offers a novel perspective on the quantum effect of polymeric insulators in organic semiconductors, but also presents a simple yet effective method for enhancing the performance of organic solar cells.

## Teaser

This work offers a novel perspective on the quantum effect of polymeric insulators in organic semiconductors and presents a simple yet effective method for enhancing the performance of organic solar cells.

## INTRODUCTION

Polymeric insulators have achieved great success in the field of organic semiconductors [1,2]. Several research groups have demonstrated favorable impacts of low-dosage insulators on the regulation of molecular aggregation, enhancement of crystallinity and trap dilution effects [3–9]. More encouragingly, Blom *et al*. discovered that the insulator poly(vinylcarbazole) (PVK) can completely passivate electron traps and tune the diluted organic semiconductor to an ideal bipolar transport case, even with an extremely high weight content (up to 90%) [1]. Generally, the blending of insulating materials has been proven to be an effective method to improve the electrical conductivity and defect characteristics of organic semiconducting thin films.

Organic solar cells (OSCs) are a more complex case [10]. Within the photoactive layer, donor and acceptor establish distinct hole and electron transport pathways, and OSCs can potentially achieve optimal performance only when charge carriers reach a state of equilibrium transport [11–14]. This implies that blending insulators in OSCs may not be as feasible as other organic electronic devices, because the dilution effect for de-trapping a specific type of carrier can lead to uncontrollable consequences for the other [1]. Due to such highly disordered nature of OSCs [15,16], the mechanism of insulator in donor:acceptor (D:A) blending systems is still unclear. There are many case studies on insulator blending in OSC systems. Incorporating insulators can significantly prolong the film formation process, improving molecular crystallinity and reducing trap state density [2,4]. Moreover, enhanced non-covalent molecular interaction through insulator blending optimizes molecular stacking, thereby boosting charge carrier delocalization and improving performance of OSCs [7–9]. Current studies show that the improvement in morphology appears to be an inevitable outcome of insulators. However, this morphological evolution cannot be theoretically correlated with the charge carrier behavior in the active layer or experimentally summarized in statistical models. Therefore, elucidating the mechanism of insulator on the active layer morphology and seeking other potential functions beyond morphological regulation are of great research and practical significance for further enhancing the performance of OSCs.

Herein, we propose an insulator-donor electron wavefunction coupling effect, which is different from the conventional understanding of the morphological control. This coupling effect can significantly enhance electron mobility (up to 100 times) through the incorporation of polymeric insulators in a series of donor systems, involving PM6, PBDB-T, PTB7-Th and PBDB-T-SF. Furthermore, inferior electron transport properties in the PM6:PE films can be observed with improved thin film crystallinity, and this phenomenon excludes the dominant effect of morphology change in electron transport evolution. The extended Su–Schrieffer–Heeger (SSH) tight-binding model indicates that the polymeric insulators with electron-rich groups can couple with the D unit of D-A type donor polymers, and thus effectively reduce the intramolecular energy barriers of electron carriers. This quantum effect can partially address the inferior electron transport behaviors due to the adverse interfacial contact in pseudo-bilayer OSCs. By applying this polymeric insulator blending strategy, we achieved a power conversion efficiency (PCE) of 19.50% (certificated 19.18%) in the PM6/L8-BO based pseudo-bilayer OSCs. This work not only offers a novel perspective on the quantum effect of polymeric insulators in organic semiconductors, but also presents a simple yet effective method for enhancing the performance of organic solar cells.

## RESULTS AND DISCUSSION

### Electron transport analysis in donor polymers

**Figure 1.**
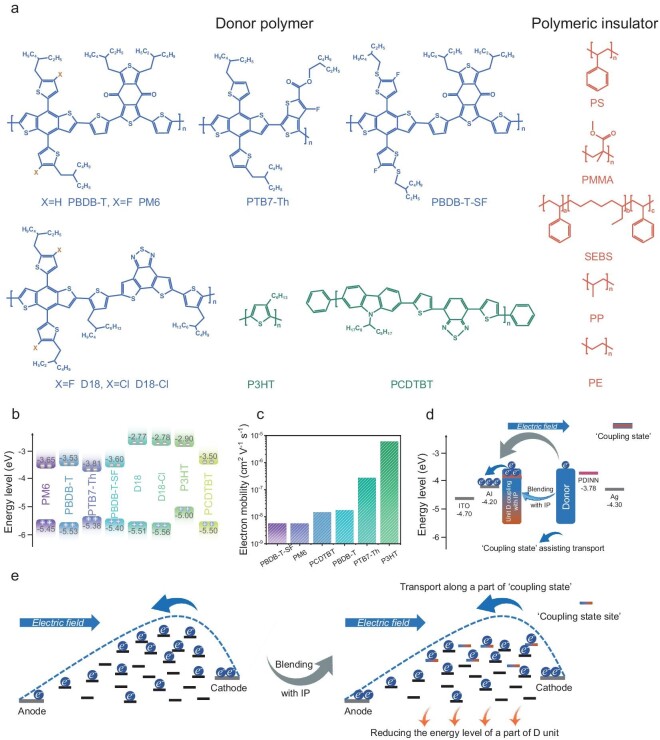
(a) The chemical structures of donor polymers and polymeric insulators in this work. (b) The energy levels of the donor polymers. (c) The neat film electron mobilities of the donor polymers. The change of electron transport behavior in donor polymer before and after blending the insulator from different angles in (d) energy level and (e) transport sites level.

Figure 1a displays the chemical structures of selected donor polymers and polymeric insulators in this work. Figure 1b and Fig. S1 depict the energy levels and normalized UV-vis absorption spectra of donor polymers. The space-charge-limited current (SCLC) model was performed to extract charge carrier mobilities [17,18]. Figure 1c summarizes the electron mobility of neat PM6, PBDB-T, PTB7-Th, PBDB-T-SF, P3HT and PCDTBT films. However, it is worth noting that the analysis of electron transport in D18 and D18-Cl films is unavailable due to the large electron injection barriers in such materials [19,20]. It can be clearly observed that the electron mobility values in donor polymers are much lower than hole cases [21]. Such unipolar charge carrier transport properties result in high charge recombination rate, hindering further decrease of non-radiative energy loss in pseudo-bilayer OSCs. Therefore, boosting the delocalization of electron and hole carriers in donor polymers may be expected to enhance the performance of OSCs [22,23]. Figure 1d and e illustrate the changes of electron transport behavior after insulator blending, and the detailed interpretation is described in a later section.

**Figure 2.**
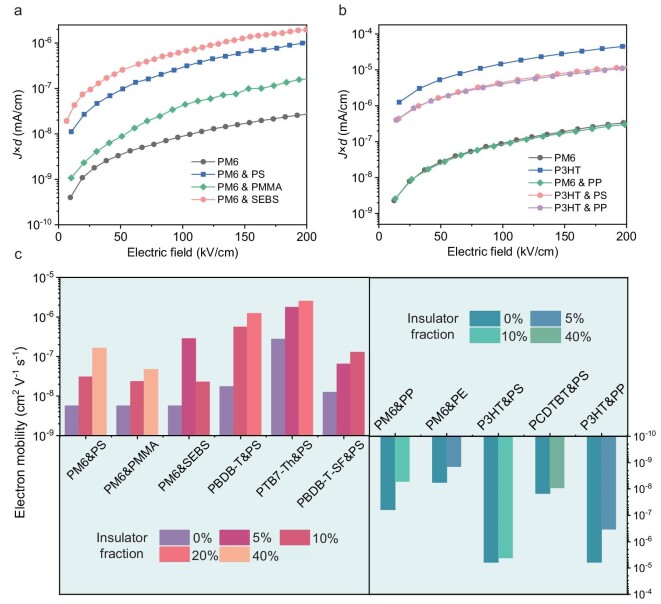
Electron transport analysis from SCLC measurements. (a, b) The dependence of *J* × *d* values with the applied electric field for different neat and blending systems. (c) Electron mobility values of donor polymers with different weight fraction polymeric insulators.

We initially chose one of the most widely-used donor PM6 with polystyrene (PS) as the model system to investigate the electron transport properties. The electron mobility ${{\mu }_e}$ of neat PM6 film is 5.7 × 10^−9^ cm^2^ V^−1^ s^−1^, which is consistent with reported results in the literature [21]. Figure S3a summarizes ${{\mu }_e}$ in PM6-based films with various PS contents; ${{\mu }_e}$ can be improved to exceed 10^−7^ cm^2^ V^−1^ s^−1^ when 40 wt% PS was blended into the PM6 film. Other donor systems exhibit similar results when we added different polymeric insulators into neat films. Figure S3 shows the variation of ${{\mu }_e}$ in PM6&PMMA, PM6&SEBS, PBDB-T&PS, PTB7-Th&PS and PBDB-T-SF&PS systems. When the polymeric insulators reach high blending fraction, μ*_e_* begins to show a downward tendency. This is due to the fact that high insulator blending fraction severely disrupts the film morphology, leading to the decay of transport network and electron mobility in donors. However, the blending of polymeric insulators cannot improve the electron transport properties in selecting donor D18, D18-Cl, P3HT, PCDTBT or insulator PP, PE cases (Figs S5 and S6). For D18 and D18-Cl, the large electron injection barrier make it is inappropriate to analyze electron transport behavior using the SCLC model. Figure 2 summarizes the current density response as a function of the applied electric field in a series of donor polymer-based electron-only devices and mobility values. It is clearly observed that the positive effect of polymeric insulators in electron transport properties only occurs in D-A type donor polymers, and the insulating component should involve electron-rich groups, such as PS, PMMA and SEBS.

**Figure 3.**
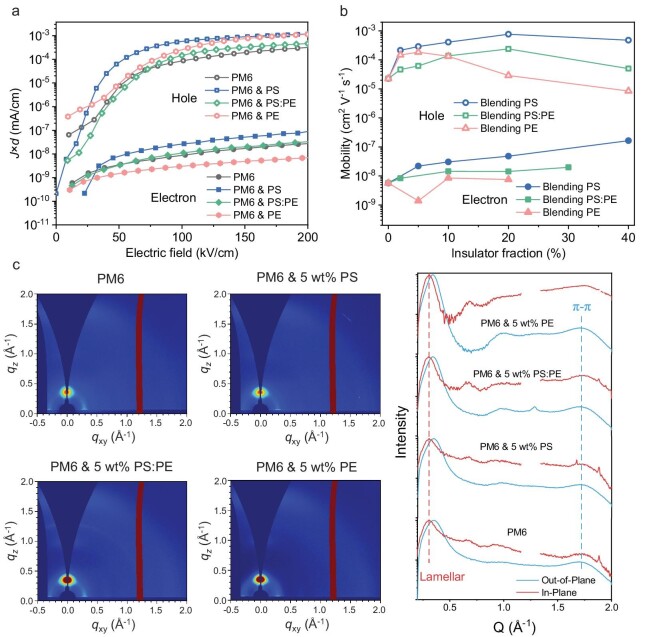
(a) The electron and hole *J* × *d* values with the applied electric field for different neat and blending systems using SCLC analysis. (b) The detailed relationship of hole and electron mobility with insulator fraction in donor PM6. (c) 2D and 1D GIWAXS patterns of PM6 and PM6 blending with insulator systems.

Next, we selected two polymeric insulators PS and PE to evaluate the relationship between the variation in electron mobility and the morphological evolution in thin films. Figure 3a shows the current density response as a function of the applied electric field in PM6-based hole-only and electron-only devices with ≤5 wt% PS, PE, and PS&PE (1:1 in weight), and Fig. 3b summarizes the mobility values as a function of polymeric insulator contents. With low dosage (<10 wt%) polymeric insulators, hole mobility ${{\mu }_h}$ values increase in all three cases, whereas ${{\mu }_e}$ values only remarkably increase in the PM6:PS system. With 5 wt% PS, ${{\mu }_e}$ is 2.2×10^−8^ cm^2^ V^−1^ s^−1^, which is ∼3.9 times higher than the neat PM6 film. In contrast, the 5 wt% PE blended film shows a low ${{\mu }_e}$ of 1.4×10^−9^ cm^2^ V^−1^ s^−1^. We further performed grazing incidence wide-angle X-ray scattering (GIWAXS) to investigate the origin of these transport behavior differences of hole and electron carriers in PS, PE and PS&PE systems. Figure 3c shows the 2D and 1D GIWAXS morphology information of PM6-based thin films with/without 5 wt% PS, PE and PS&PE materials. Figure S7 and Table S1 summarize parameters of π–π stacking information in the out-of-plane direction. The π–π stacking interaction and crystallinity of all thin films can be improved with polymeric insulators, and the π–π distance/crystalline coherence length (CCL) after blending 5 wt% PS, PE and PS&PE are 3.61 Å/19.26 Å, 3.63 Å/18.87 Å, 3.64 Å/17.93 Å, respectively, while that of the PM6 neat film is 3.65 Å/17.38 Å. The morphology evolution shows a high correlation with the change of hole mobilities. However, the variation of electron mobility mismatches with the thin film morphology with the blending of polymeric insulators, and therefore, it is implausible to only attribute the electron behavior with the morphological change. Therefore, we can conclude that the variations of electron and hole mobilities after polymeric insulator blending are driven by distinct mechanisms. Specifically, hole mobility is more closely linked to morphology, whereas the changes of electron mobility may also be driven by potential effects beyond morphology, which eventually lead to different tendencies for the electron and hole transport behavior after insulator blending.

Based on the analysis above, we can conclude that two essential prerequisites may contribute to the enhancement of electron transport property in insulator-donor blending films: (1) the side chains of polymeric insulators should contain electron-rich units such as a benzene ring; and (2) the donor polymer should contain D-A repeating units, meaning the push electron unit and the pull electron unit appear alternately in the polymer backbone. To elucidate the mechanism for the enhancement of electron transport properties when polymeric insulators are blended into donor polymers, we concurrently analyze the structural characteristics of above specific donor polymers and insulators. One possible reason is that after blending the polymeric insulator containing the electron-rich group with the D-A polymers, the insulator may couple with the D or/and A unit of the polymer, and thereby reduce the barrier height of the D unit or the potential well depth of the A unit in the polymer backbone, and thus facilitate intramolecular electron transport.

### Molecular dynamics and tight-binding model simulations

In order to elaborate the mechanism of insulator-donor electron wavefunction coupling effect in improving electron mobility, we performed molecular dynamic (MD) and tight-binding model simulations. For the MD simulation, we conducted PM6 and PM6:Y6 films with various PS contents. The radial distribution function and numbers of molecule pairs in close proximity (<6 Å) between D or A unit and PS are summarized in Fig. S8 and Table S2. We can observe that in both the neat donor and blending cases, PS molecules can interact with PM6. Moreover, the number of PS molecules near the D and A units is similar, indicating that they may have an equal probability of interacting with the D or A units.

**Figure 4.**
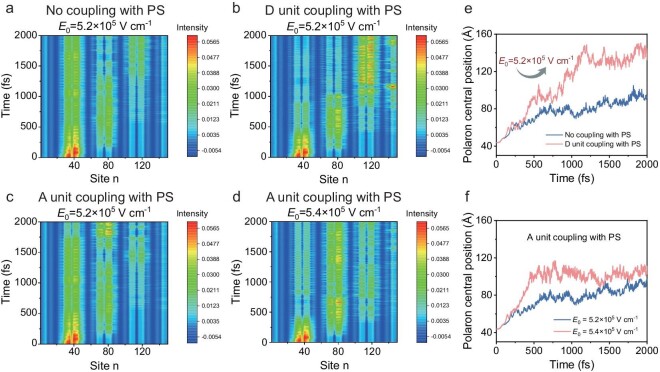
Time evolutions of the net charges on each site of the corresponding molecular models. (a) No coupling with PS, (b) D unit coupling with PS and (c) A unit coupling with PS under *E*_0_ = 5.2 × 10^5^ V cm^−1^, (d) A unit coupling with PS under *E*_0_ = 5.4 × 10^5^ V cm^−1^. Time evolutions of the charge center positions of negative polaron of the corresponding molecular models. (e) No coupling with PS and D unit coupling with PS under *E*_0_ = 5.2 × 10^5^ V cm^−1^. (f) A unit coupling with PS under *E*_0_ = 5.2 × 10^5^ V cm^−1^ and 5.4 × 10^5^ V cm^−1^.

The extended Su–Schrieffer–Heeger (SSH) tight-binding model was employed to accurately elucidate the electron transport behavior in donor polymer [24]. We established a polymer model which has four-polymerized units (*n* = 4) and its corresponding molecular models in different coupling situations with insulator PS. For the coupling effect, we considered that the carbon atoms between PS side chain benzene ring and donor polymer thiophene ring has an electronic transition integral. Detailed modeling information has been described in the Supporting Information. We assumed that a negative polaron was initially generated at the left side of the polymer chain. The initial net charge density distribution curve of each grid point is shown in Fig. S9. This distribution indicates that there is an intramolecular charge transfer character, caused by the different on-site energy of D and A units, and it also implies that D units will act as a potential barrier to impede electron transport [25,26]. As demonstrated in Fig. S10, the negative polaron in the polymer chain can transport from the initial position to the right end group, at least under the electric field strength of *E*_0_ = 5.2×10^5^ V cm^−1^ for no coupling with PS, *E*_0_ = 5.1×10^5^ V cm^−1^ for D unit coupling with PS and *E*_0_ = 5.4×10^5^ V cm^−1^ for A unit coupling with PS. Therefore, we initially conclude that PS coupling with polymer A unit is not conducive for intrachain electron transport due to large threshold electric fields. Figure 4a–c illustrate the time evolutions of net charges on each site for three coupling cases under *E*_0_ = 5.2×10^5^ V cm^−1^, while Fig. 4d shows the case of A unit coupling with PS under *E*_0_ = 5.4×10^5^ V cm^−1^. We observed that after applying the identical electric field *E*_0_ = 5.2×10^5^ V cm^−1^ for 2000 fs, the negative polaron has already reached the right end group in the case of D unit coupling with PS. Figure 4e and f show corresponding time evolution of charge center position for the negative polaron. To intuitively obtain the transport velocities of electron polaron under two cases: no coupling with PS and D unit coupling with PS, we calculated the average transport velocity, 0.342 nm fs^−1^ for no coupling with PS and 0.852 nm fs^−1^ for D unit coupling with PS. The calculation results also tend to support that D unit coupling with PS is favorite for electron transport, consistent with the phenomena observed in the experiment. In short, we find that insulators containing electron-rich group can couple with D unit of D-A donor polymer, effectively reducing the energy barrier, which is conducive for polymer intrachain electron transport. The non–D-A type polymers themselves do not contain an alternating energy level structure, and thus this coupling effect cannot be applied.

Figure 1d and e visualizes this transport mechanism at different angles. From the point of view of energy level, Fig. 1d describes that after the insulator electron-rich group coupling with D unit, a coupling state is provided, which can effectively reduce the energy barrier of the D unit in a polymer chain. Therefore, this coupling state is a conducive path for electron transport. Figure 1e graphically shows the variation of state sites. When insulator coupling with donor, some coupling state sites can be provided, which have lower barriers and are beneficial for the electron transport process.

### Device fabrication of pseudo-bilayer organic solar cells

Pseudo-bilayer OSCs have attracted extensive attention due to favorable vertical phase separation and sufficient exciton dissociation interfaces [27–37]. However, the penetration of acceptors attributed to the diffusion and swelling effect in the two-step donor/acceptor deposition process presents a double-edged sword. This uneven diffusion and swelling not only give rise to interpenetrating networks but also generate acceptor-isolated domains in the donor phase. Therefore, trapped free electrons within the isolated domains inevitably undergo high recombination rates, which decrease the overall performance of pseudo-bilayer OSCs.

**Figure 5.**
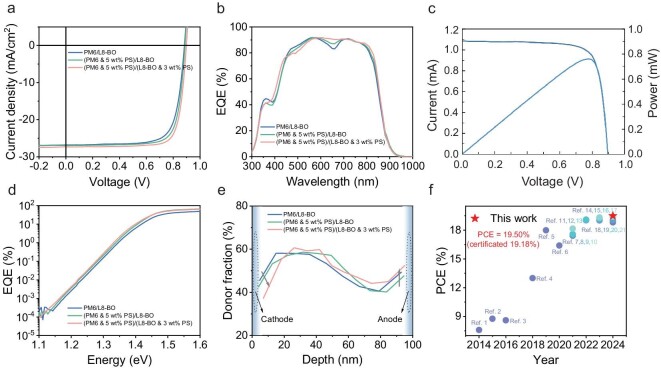
(a) *J*–*V* curves of the pseudo-bilayer OSCs based on PM6/L8-BO, (PM6 & 5 wt% PS)/L8-BO and (PM6 & 5 wt% PS)/(L8-BO & 3wt% PS) systems under AM 1.5 G conditions. (b) EQE spectra. (c) Certificated *I*-*V* curve for a (PM6 & 5 wt% PS)/(L8-BO & 3 wt% PS) device. (d) Sensitive EQE spectra. (e) Vertical phase distribution of the corresponding pseudo-bilayer OSCs. (f) Summary of PCEs for the pseudo-bilayer OSCs from literature and this work. (The corresponding references are listed in the Supporting Information.)

Based on the above understanding, we selected PS to regulate the electron transport behavior in the donor layer of the PM6/L8-BO device, aiming to fully utilize the free electrons generated at the interface of isolated domains. The PM6/L8-BO device achieved a PCE of 17.90%, while the (PM6 with 5 wt% PS)/L8-BO device exhibited an enhanced PCE of 18.67%. The corresponding *J*-*V* and EQE curve are shown in Fig. 5a and b, and the device parameters are summarized in Table 1 and Table S3. To comprehensively apply the insulator blending strategy for both morphology regulation [2] and electron delocalization, we fabricated a pseudo-bilayer (PM6 with 5 wt% PS)/(L8-BO with 3 wt% PS) device yielding a record PCE of 19.50% (certificated 19.18%, as shown in Fig. 5c and Fig. S11), which to the best of our knowledge is one of the highest certificated PCEs for pseudo-bilayer devices to date (Fig. 5f and Table S4).

**Table 1.** Photovoltaic performance parameters of the pseudo-bilayer OSCs under AM 1.5 G conditions.


System	*V*_oc_ (V)	*J*_sc_ (mA/cm^2^)	*J*_cal_ (mA/cm^2^)	FF (%)	PCE (%)
PM6/L8-BO	0.883	26.81	25.92	75.5	17.90 (17.55 ± 0.37)
(PM6 & 5 wt% PS)/L8-BO	0.888	26.96	26.14	78.0	18.67 (18.30 ± 0.28)
(PM6 & 5 wt% PS)/(L8-BO & 3 wt% PS)	0.898	27.32	26.39	79.5	19.50 (19.22 ± 0.27)
(PM6 & 5 wt% PS)/(L8-BO & 3 wt% PS) (Certificated)	0.892	27.44	–	78.4	19.18

To elucidate the function of polymeric insulators in pseudo-bilayer OSCs, we conducted comprehensive characterization of the above devices from multiple perspectives. The electron hopping distance can be expressed as $\mu = e\nu {{R}^2}/6kT$, where *R* is average hopping distance, and *ν* is jump frequency [38,39]. After blending 5 wt% PS, the electron hopping ability increases from 1.72 Å to 3.37 Å. The UV-vis absorption spectrum of the corresponding systems is depicted in Fig. S12. The redshift of the acceptor absorption peak can be observed in the (PM6 with 5 wt% PS)/(L8-BO with 3 wt% PS) system, indicating enhanced molecular stacking of L8-BO in the acceptor layer. Additionally, we performed sensitive sub-bandgap external quantum efficiency (s-EQE) techniques to investigate the effect of blending PS on the tail state (Fig. 5d and Table S5). Compared with the control device of 24.9 meV, the (PM6 with 5 wt% PS)/(L8-BO with 3 wt% PS) device has a lower Urbach energy (*E*_u_) value of 24.3 meV. The results show that blending PS can slightly reduce the tail state. Further insights were gained from GIWAXS to characterize the molecule stacking and ordering [40–42], as shown in Fig. S13 and summarized in Table S6. The GIWAXS results indicate that the (PM6 with 5 wt% PS)/(L8-BO with 3 wt% PS) system exhibits the largest CCL in both OOP and IP directions as the indictor for an enhanced molecular ordering consistent with the absorption peak shift and Urbach energy change [2]. To gain deeper insights from the morphology perspective, film-depth–dependent light absorption spectroscopy (FLAS) was performed to study the vertical distribution (Figs S14 and S15). The results show that more acceptors are enriched near the cathode, and more donors are enriched near the anode in the (PM6 with 5 wt% PS)/(L8-BO with 3 wt% PS) film (Fig. 5e), which is more conducive for the electron and hole transport to corresponding electrodes. In addition, from the photoluminescence (PL) spectrum in Fig. S16 and Table S7, the weakened PM6 peak strength ratio of the (PM6 with 5 wt% PS)/(L8-BO with 3 wt% PS) system suggests enhanced electron transfer from donor to acceptor [43–46]. From the electrical characteristics of charge carriers, transient photovoltage/current (TPV/TPC) and photo-induced carrier extraction by linearly increasing voltage (photo-CELIV) testing were used to investigate transport and recombination parameters as shown in Fig. S17. The PM6 blending with PS system exhibits favorable carrier lifetime, extraction time and mobility values. The synergistic effects of the above mechanisms cooperatively promote the dissociation of excitons and the extraction of free carriers, ultimately achieving a high efficiency of PS-blended pseudo-bilayer OSCs.

## CONCLUSIONS

In this work, we propose a novel mechanism for the polymeric insulator blending effect: an insulator-donor electron wavefunction coupling effect. Polymeric insulators containing electron-rich group side chains can effectively improve the electron mobility of D-A donor polymers, achieving a surprising increase of electron mobilities in donor polymers up to nearly 100 times. The polymeric insulators coupling with D unit of donor polymers can reduce the barrier height and effectively improve electron transport. This mechanism can be used to regulate the electron transport of pseudo-bilayer OSCs. We fabricated a pseudo-bilayer (PM6 with 5 wt% PS)/(L8-BO with 3 wt% PS) device yielding a record PCE of 19.50% (certificated 19.18%), which to the best of our knowledge is one of the highest certificated PCEs for pseudo-bilayer devices to date. This work not only offers a novel perspective on the quantum effect of polymeric insulators in organic semiconductors, but also presents a simple yet effective method for enhancing the performance of organic solar cells.

## Supplementary Material

nwae385_Supplemental_File
