# Supplementary material for: Insulator-donor electron wavefunction coupling in pseudo-bilayer organic solar cells achieving a certificated efficiency of 19.18%
Source: Natl Sci Rev. 2024 Oct 30;12(1):nwae385. doi: 10.1093/nsr/nwae385 (PMC11702652; doi:10.1093/nsr/nwae385)
Supplement: nwae385_Supplemental_File [file nwae385_supplemental_file.zip › Supplementary data.pdf]

Supporting Information

Insulator-donor electron wavefunction coupling in pseudo-bilayer organic solar cells achieving a certificated efficiency of 19.18%

Jiangkai Sun,[1,†] Ruijie Ma,[2,†] Xue Yang,[1,†] Xiaoyu Xie,[3] Dongcheng Jiang,[1] Yuan Meng,[1] Yiyun Li,[1] Fengzhe Cui,[1] Mengfei Xiao,[1] Kangning Zhang,[1] Yu Chen,[5] Xinxin Xia,[6] Maojie Zhang,[6] Xiaoyan Du,[1] Long Ye,[4] Haibo Ma,[3] Kun Gao,[1,*] Feng Chen,[1] Gang Li,[2,*] Xiaotao Hao,[1,*] and Hang Yin,[1,*]

[1]School of Physics, State Key Laboratory of Crystal Materials, Shandong University, Jinan 250100, China;

[2]Department of Electric and Electronic Engineering, Research Institute for Smart Energy (RISE), Photonic Research Institute (PRI), The Hong Kong Polytechnic University, Hong Kong 999077, China;

[3]Qingdao Institute for Theoretical and Computational Sciences, School of Chemistry and Chemical Engineering, Shandong University, Qingdao 266237, China;

[4]School of Materials Science and Engineering, Tianjin Key Laboratory of Molecular Optoelectronic Sciences, Collaborative Innovation Center of Chemical Science and Engineering (Tianjin), Tianjin University, Tianjin 300350, China;

[5]Beijing Synchrotron Radiation Facility, Institute of High Energy Physics, Chinese Academy of Sciences, Beijing 100049, China;

[6]National Engineering Research Center for Colloidal Materials, School of Chemistry and Chemical Engineering, Shandong University, Jinan 250100, China

***Corresponding authors.** E-mails: gk@sdu.edu.cn; gang.w.li@polyu.edu.hk; haoxt@sdu.edu.cn; hyin@sdu.edu.cn

[†]Equally contributed to this work.

Keywords: organic semiconductors, polymeric insulators, electron transport, organic solar cells

Materials

Organic photovoltaic materials PM6, PBDB-T, PTB7-Th, PBDB-T-SF, P3HT, L8-BO, PDINN used in this work were purchased from Solarmer Materials Inc. D18 and D18-Cl were purchased from eFlexPV Limited. PCDTBT was purchased from 1-Material. PS, PMMA, PP were purchased from Sigma-Aldrich Corporation. PE was purchased from Sigma Materials (Shanghai) Inc. The polymer elastomer SEBS was available from Asahi Kasei. PEDOT:PSS (Clevios PVP Al 4083) was purchased from Xi'an Polymer Light Technology Corp. Chloroform, methanol, 1,8-Diiodooctane (DIO) were purchased from Sigma Aldrich Inc. All materials were used without further purification.

Single-carrier device fabrication and characterization

The electron-only devices were fabricated with a conventional configuration of ITO/Al(50nm)/Active layer/PDINN/Ag. The patterned indium tin oxide (ITO)-coated substrates were sequentially cleaned in an ultrasonic bath by using detergent, deionized water, acetone, absolute ethyl alcohol and isopropyl alcohol for 20 min in each step. These glass substrates were then treated by UV-ozone for 15 min to improve their work function. The 50 nm Al layer deposited onto these cleaned substrates by thermal evaporation is used to block hole carriers. Afterwards, these prepared substrates were transferred into a nitrogen-filled glove-box. The solution can be obtained by dissolving an organic donor polymer and insulating polymer material through organic solvent such as chloroform (CF). The active layer films (~200 nm) were fabricated by spin-coating the solution on the top of Al and then annealed. After that, a thin layer of PDINN was spin coated on BHJ layer to contribute to the electron transport at 3000 rpm for 30 s. Finally, the top Ag electrodes of 100 nm were deposited on the top of PDINN layer by thermal evaporation to finish the preparation of the whole devices, thus yielding the active area of 0.024 cm^2 through a shadow mask. The hole-only devices were fabricated with the structure of ITO/PEDOT:PSS/Active layer/Spiro-TPD/Au. The PEDOT:PSS was spin-coated onto

these cleaned substrates at 4000 rpm for 50 s and annealed at 150 ℃ for 15 min in ambient atmosphere. Afterwards, these prepared substrates were transferred into a nitrogen-filled glove box. The active layer films (~200 nm) were fabricated by spin-coating the blend solution on the top of PEDOT:PSS layer and then annealed. After a 10 nm Spiro-TPD layer deposited by thermal evaporation, an 80 nm Au layer was deposited on the substrate. The current density-voltage (*J-V*) characteristic curves of all devices were recorded in a low vacuum environment by employing a computer-controlled Keithley 2612B.

GIWAXS measurements

The GIWAXS date was obtained at 1W1A Diffuse X-ray Scattering Station, Beijing Synchrotron Radiation Facility (BSRF-1W1A). Samples were prepared on silicon substrates using identical blend solutions as those used in devices.

The Calculation of Crystalline Coherence Length

GIWAXS characterizes the molecular packing and crystallinity of the active layer. The *d*-spacing associated with the π-π stacking peak indicates the molecular interlayer spacing, which can be calculated from the following equation:

$$d = \frac{2\pi}{q_{z(010)}}$$

In which, $q_{z(010)}$ is the position of the π-π stacking peak in Q space. The results indicate that the π-π stacking spacings of molecules in the (010) direction are comparable in all films. We further calculated the crystalline coherence length (CCL) using the Scherrer equation and quantitatively compared the crystallinity of the films:

$$CCL = \frac{2\pi k}{FWHM}$$

In which, k is the shape factor (0.9) and $FWHM$ is the full width at half maximum of the π-π stacking peak.

FTPS measurements

The FTPS-EQE measurements were performed by employing a Fourier-transform infrared (FTIR) spectrometer equipped with a halogen lamp light source and an external detector option. The photocurrent generated from the devices with illumination light modulated by the FTIR instrument was amplified by a low-noise current amplifier. The corresponding photocurrent spectrum were recorded by the FTIR software.

Film-depth-dependent light absorption spectroscopy (FLAS)

The film-depth-dependent light absorption spectroscopy was acquired upon a film-depth-dependent light absorption spectrometer (PU100, Puguangweishi Co. Ltd). In-situ soft plasma etching at low pressure (less than 20Pa) was used to extract the depth-resolved absorption spectrum for the organic active layer. Beer-Lambert's law was utilized to fit the FLAS results, which were subsequently utilized to fit the exciton generation contour upon a modified optical matrix-transfer approach.

The Measurements of Photoluminescence (PL)

The PL spectra was acquired through a confocal optical microscope (Nanofinder FLEX2, Tokyo Instruments, Inc.) equipped with time-correlated single-photon counting (TCSPC) module (Becker & Hickl, SPC-150). All of the PL spectra were measured using a charge-coupled device (CCD) sensor (DU420A-OE, Andor). The excitation wavelength was fixed at 400 nm. The excitation power of all the PL spectra and was fixed at 1 μW.

The tight-binding model and nonadiabatic evolution method

Herein, a detailed description is presented for the three models, including the single polymer, the unit D of the polymer coupling with PS and the unit A of the polymer coupling with PS. For a polymer chain, it is constructed of four repeat units (j=4) and three linker units (k=3). Every repeat unit contains one electron-push central fused ring group and two electron-pull terminal groups, forming a linear conjugated

backbone with the well-known A-D-A type electronic structure.

The sites of the polymer chain are uniformly numbered as n=1→N for the first repeat unit, n=N+1→N+L for the first linker unit, n=N+L+1→2N+L for the second repeat unit, 2N+L+1→2N+2L for the second linker unit, n=2N+2L+1→3N+2L for the third repeat unit, n=3N+2L+1→3N+3L for the third linker unit, and n=3N+3L+1→4N+3L for the fourth repeat unit, where N=36 indicates the total site number of a repeat unit, L=2 indicates the total site number of a linker unit, the total site number of a single polymer chain is N_1=4N+3L=150.

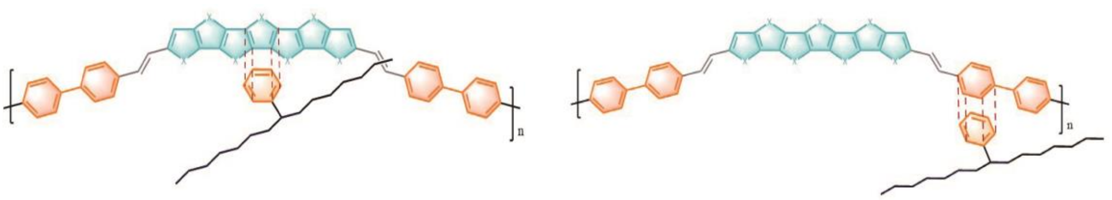

D unit coupling with PS **A unit coupling with PS**

Considering the coupling effect, we assume that there were four carbon atoms on benzene ring of PS side chain coupled with its corresponding atoms on the central thiophene ring of D unit (or benzene ring of the A unit) of polymer, for the second and the third repeat unit respectively (the diagram only shows the coupling within a repeating unit). The sites of benzene ring on PS are uniformly numbered as n=$N_1 + 1$→$N_1 + 4$ for the first benzene ring and n=$N_1 + 5$→$N_1 + 8$ for the second benzene ring.

The total Hamiltonian of the polymer (H_{P4}) with no coupling is written as

$$H_{P4} = \sum_{j=1}^{4} H_{A_j} + \sum_{k=1}^{3} H_{link_k} + \frac{K}{2}\sum_{n}(u_{n+1} - u_n)^2 + \frac{M}{2}\sum_{n}\dot{u}_n^2$$

H_{A_j} $(j = 1,2,3,4)$ is the Hamiltonian for the repeat unit, described as

$$H_{A_j} = \sum_{n=(j-1)(N+L)+1}^{jN+(j-1)L-1} t_{j,n,n+1}(C_{j,n+1,s}^{+}C_{j,n,s} + C_{j,n,s}^{+}C_{j,n+1,s})$$
$$+ t_3 \sum_{l=1}^{4} (C_{j,(j-1)(N+L)+4l+7,s}^{+}C_{j,(j-1)(N+L)+4l+10,s}$$
$$+ C_{j,(j-1)(N+L)+4l+10,s}^{+}C_{j,(j-1)(N+L)+4l+7,s})$$
$$+ t_3 \sum_{l=1}^{3} (C_{j,(j-1)(N+L)+4l+9,s}^{+}C_{j,(j-1)(N+L)+4l+12,s}$$
$$+ C_{j,(j-1)(N+L)+4l+12,s}^{+}C_{j,(j-1)(N+L)+4l+9,s}) + \sum_{n'} \Delta_{on}(n')C_{j,n'}^{+}C_{j,n'}$$
$$- \Delta'_{on} \sum_{n''} C_{j,n''}^{+}C_{j,n''}$$

$t_{j,n,n+1}$ represents the electron hopping integral between the nearest-neighbor sites, described as

$$t_{j,n,n+1} = \begin{cases} t_{n,n+1}^{T}, & 1 + (j-1)(N+L) \le n \le 7 + (j-1)(N+L), \\ & 29 + (j-1)(N+L) \le n \le 35 + (j-1)(N+L) \\ t_{n,n+1}^{\pi}, & 8 + (j-1)(N+L) \le n \le 10 + (j-1)(N+L), \\ & 26 + (j-1)(N+L) \le n \le 28 + (j-1)(N+L) \\ t_{n,n+1}^{C}, & 11 + (j-1)(N+L) \le n \le 25 + (j-1)(N+L) \end{cases}$$

$t_{n,n+1}^{T}$, $t_{n,n+1}^{\pi}$, $t_{n,n+1}^{C}$ are described as follows:

$$t_{n,n+1}^{T} = t_0 - \alpha(u_{n+1} - u_n) - t_1\cos(n\pi/2)$$
$$t_{n,n+1}^{\pi} = t_0 - \alpha(u_{n+1} - u_n)$$
$$t_{n,n+1}^{C} = t_0 - \alpha(u_{n+1} - u_n) - (-1)^n t_2$$

For any repeat unit, $C_{j,n,s}^{+}(C_{j,n,s})$ denotes the creation (annihilation) operator of an electron with spin s (s=↑,↓) at site n, u_n is the displacement of site n. t_0 represents the nearest-neighbor hopping integral for a uniform molecular bond structure, t_1 and t_2 are the symmetry-breaking parameters, separately introduced to describe the lattice feature of terminal groups (t_1) and central group (t_2) of each

repeat unit, α represents the e-l interaction constant. $\sum_{n'}$ means the summation only for the sites on the central groups and $\sum_{n''}$ means the summation only for the sites on the terminal groups. To further consider the effect of heteroatoms X (e.g., S and N atoms) on the central group, we introduce t_3 to describe the electron hopping between the neighboring sites of a heteroatom. Furthermore, we introduce the on-site energy Δ'_{on} to describe the fluorinated or chlorinated contribution to the electron-pull ability of the terminal groups, and the on-site energy $\Delta_{on}(n')$ to describe the heteroatom contribution to the electron-push ability of the central group, $\Delta_{on}(n')$ on each central group is a Gaussian form centered at site n_C=18.5 with a width n_w=4, written as

$$\Delta_{on}(n) = \begin{cases} \Delta_{on_0}\exp[-(n-n_C)^2]/{n_w}^2, & 11 \leq n \leq 29 \\ \Delta_{on}(n-N-L), & N+L+11 \leq n \leq N+L+29 \\ \Delta_{on}(n-2N-2L), & 2N+2L+11 \leq n \leq 2N+2L+29 \\ \Delta_{on}(n-3N-3L), & 3N+3L+11 \leq n \leq 3N+3L+29 \end{cases}$$

H_{link_k} $(k=1,2,3)$ is the Hamiltonian for the linker unit, described as

$$H_{link_k} = -\sum_{n=kN+(k-1)L}^{k(N+L)} t^{link}_{k,n,n+1}(C^+_{k,n+1,s}C_{k,n,s} + C^+_{k,n,s}C_{k,n+1,s})$$

$t^{link}_{k,n,n+1}$ represents the electron hopping integral between the nearest-neighbor sites, described as

$$t^{link}_{k,n,n+1} = t_0 - \alpha(u_{n+1} - u_n)$$

Similarly, for any linker unit, $C^+_{k,n,s}(C_{k,n,s})$ denotes the creation (annihilation) operator of an electron with spin s (s=↑,↓) at site n, u_n is the displacement of site n.

The last two terms are the lattice part, describing the elastic potential energy and the kinetic energy. K represents the elastic constant and M the mass of a site.

For case of D(A) unit coupling with PS, the Hamiltonian of the system consists of the polymer part (H_{P4}, as mentioned earlier), the PS part (H_{PS}) and the coupling part ($H_{CD(CA)}$).

$$H = H_{P4} + H_{PS} + H_{CD(CA)}$$

H_{PS} is described as

$$H_{PS} = -\sum_{n=151}^{153} t^{ps}_{n,n+1}(C^{+}_{n+1}C_n + C^{+}_{n}C_{n+1}) - \sum_{n=155}^{157} t^{ps}_{n,n+1}(C^{+}_{n+1}C_n + C^{+}_{n}C_{n+1})$$
$$- \sum_{n=151}^{158} \Delta''_{on} C^{+}_{n}C_n + \frac{K}{2}\sum_{n=151}^{153}(u_{n+1} - u_n)^2 + \frac{K}{2}\sum_{n=155}^{157}(u_{n+1} - u_n)^2$$
$$+ \frac{M}{2}\sum_{n=151}^{158} \dot{u}^2_n$$

$t^{ps}_{n,n+1}$represents the electron hopping integral between the nearest-neighbor sites on benzene ring of PS side chain, described as

$$t^{ps}_{n,n+1} = t_0 - \alpha(u_{n+1} - u_n) - t_1\cos(n\pi/2)$$

Notably, $t^{ps}_{154,155} = t^{ps}_{155,154} = 0$, because the 154th point and the 155th point belong to different molecules. Furthermore, the on-site energy Δ''_{on} is introduced to describe the electron-pull ability of the benzene ring of PS side chain.

H_{CD} is the coupling part under the case of D unit coupling with PS, described as

$$H_{CD} = -t_{\perp}\sum_{n=55}^{58}(C^{+}_{n+96}C_n + C^{+}_{n}C_{n+96}) - t_{\perp}\sum_{n=93}^{96}(C^{+}_{n+62}C_n + C^{+}_{n}C_{n+62})$$

H_{CA} is the coupling part under the case of A unit coupling with PS, described as

$$H_{CA} = -t_{\perp}\sum_{n=71}^{74}(C^{+}_{n+80}C_n + C^{+}_{n}C_{n+80}) - t_{\perp}\sum_{n=109}^{112}(C^{+}_{n+46}C_n + C^{+}_{n}C_{n+46})$$

$t_{\perp} = \frac{t_0}{10}\exp[1 - d/5]$ indicates the inter-molecular electron hopping integral between the vertical neighbor sites of polymer and benzene ring on the side chain of PS, determined by the inter-molecular distance d (d=3 Å).

Now, let us clarify how we get the initial state before dynamical simulations. Placing an electron to the lowest unoccupied molecular orbital level (LUMO), we can get a negative polaron in the polymer by iteratively solving the static electronic

eigenequation and the lattice balance equation.

For the polymer, the static electronic eigenequation is described as

$$t_3\phi_{v,s}(i+3)[\delta(\frac{i-(j-1)L-7}{4},int)+\delta(\frac{i-(j-1)L-9}{4},int)]+t_3\phi_{v,s}(i-3)[\delta(\frac{i-(j-1)L-10}{4},int)+\delta(\frac{i-(j-1)L-12}{4},int)]+\Delta_{on}(i)\phi_{v,s}(i)-\Delta'_{on}\phi_{v,s}(n')-t_{j,n,n+1}\phi_{v,s}(n+1)-t_{j,n,n-1}\phi_{v,s}(n-1)=\varepsilon_v\phi_{v,s}(n)$$

i represents the site index of central groups, and the j represents the jth repeat unit which the ith point belongs. δ(x,int)=1, if x=int; and δ(x,int)=0, if x≠int, where "int" means an integer.

the lattice balance equation is described as

$$u_{n+1}-u_n=-\frac{2\alpha}{K}(\rho_{n,n+1}-\frac{1}{(N_1-1)}\sum_{n=1}^{N_1-1}\rho_{n,n+1})$$

N_1 is the total site number of a polymer molecule.

we can obtain the lattice balance equation by minimizing the total energy of the molecule.

For benzene ring on the side chain of PS, the static electronic eigenequation is described as

$$-t_{n,n+1}\phi_{v,s}(n+1)-t_{n,n-1}\phi_{v,s}(n-1)-\Delta''_{on}\phi_{v,s}(n)=\varepsilon_v\phi_{v,s}(n)$$

the lattice balance equation is described as

$$u_{n+1}-u_n=-\frac{2\alpha}{K}(\rho_{n,n+1}-\frac{1}{(N_{2(3)}-1)}\sum_{n=1}^{N_{2(3)}-1}\rho_{n,n+1})$$

$N_{2(3)}$ is the total site number of the first (second) benzene ring.

Based on the initial state of the negative polaron at the left lattice point of the molecular chain in the model, we apply an electric field E(t) along the molecular chain to drive the polaron, and the E(t) chosen as a semi-Gaussian form centered at time t_C=75 fs with a width t_w=25 fs,

$$E(t)=\begin{cases}E_0\exp[-(t-t_C)^2/{t_w}^2], & t\le t_C\\ E_0 \quad , & t>t_C\end{cases}$$

E_0 refers to the electric field strength. For the case of D unit coupling with PS, the contribution of the electric field can be described as

$$H_E = E(t)\sum_{n=1}^{150} e[(n-1)a+u_n](C_n^+C_n-\frac{1}{2})$$
$$+E(t)\sum_{n=151}^{154} e[(n-96-1)a+u_n](C_n^+C_n-\frac{1}{2})$$
$$+E(t)\sum_{n=155}^{158} e[(n-62-1)a+u_n](C_n^+C_n-\frac{1}{2})$$

While for the case of A unit coupling with PS,

$$H_E = E(t)\sum_{n=1}^{150} e[(n-1)a+u_n](C_n^+C_n-\frac{1}{2})$$
$$+E(t)\sum_{n=151}^{154} e[(n-80-1)a+u_n](C_n^+C_n-\frac{1}{2})$$
$$+E(t)\sum_{n=155}^{158} e[(n-46-1)a+u_n](C_n^+C_n-\frac{1}{2})$$

where e indicates the electronic charge and a the lattice constant (a=1.22 Å). When the E(t) is turned on, the system will experience an evolution. By using a nonadiabatic evolution method, we can separately obtain the temporal evolution of the electronic state $\Psi_{\nu,s}(n,t)$ and the lattice displacement $u_n(t)$.

Firstly, for a single polymer chain without coupling with PS, the evolution of an electronic state $\Psi_{\nu,s}(n,t)$ depends on the time dependent Schrödinger equation,

$$i\hbar\frac{\partial\Psi_{\nu,s}(n,t)}{\partial t} = -t_{n,n+1}\Psi_{\nu,s}(n+1,t) - t_{n-1,n}\Psi_{\nu,s}(n-1,t) + t_3\{\Psi_{\nu,s}(i+3,t)[\delta(\frac{i-(j-1)L-7}{4},int)+\delta(\frac{i-(j-1)L-9}{4},int)] + \Psi_{\nu,s}(i-3,t)\delta(\frac{i-(j-1)L-10}{4},int)+\delta(\frac{i-(j-1)L-12}{4},int)\} + \Delta_{on}\Psi_{\nu,s}(i,t) - \Delta'_{on}\Psi_{\nu,s}(n',t) + eE(t)[(n-1)a+u_n]\Psi_{\nu,s}(n,t)$$

$\Psi_{\nu,s}(n,t) =< n|\Psi_{\nu,s}(t) >$ is projection of electronic state $|\Psi_{\nu,s}(t) >$ on the Wannier state of site n.

Secondly, for the case of D unit coupling with PS, the evolution of an electronic

state $\Psi_{\nu,s}(n,t)$ depends on the time dependent Schrödinger equation:

For the polymer part,

$$i\hbar\frac{\partial\Psi_{\nu,s}(n,t)}{\partial t} = -t_{n,n+1}\Psi_{\nu,s}(n+1,t) - t_{n-1,n}\Psi_{\nu,s}(n-1,t) + t_3\{\Psi_{\nu,s}(i+3,t)[\delta(\frac{i-(j-1)L-7}{4},int) + \delta(\frac{i-(j-1)L-9}{4},int)] + \Psi_{\nu,s}(i-3,t)\delta(\frac{i-(j-1)L-10}{4},int) + \delta(\frac{i-(j-1)L-12}{4},int)\} + \Delta_{on}\Psi_{\nu,s}(n,t) - \Delta'_{on}\Psi_{\nu,s}(n',t) + eE(t)[(n-1)a + u_n]\Psi_{\nu,s}(n,t) - t_{\perp}[\delta(n,55) + \delta(n,56) + \delta(n,57) + \delta(n,58)]\Psi_{\nu,s}(n+96,t) - t_{\perp}[\delta(n,93) + \delta(n,94) + \delta(n,95) + \delta(n,96)]\Psi_{\nu,s}(n+62,t)$$

And for the PS part,

$$i\hbar\frac{\partial\Psi_{\nu,s}(n,t)}{\partial t} = -t_{n,n+1}\Psi_{\nu,s}(n+1,t) - t_{n-1,n}\Psi_{\nu,s}(n-1,t) + eE(t)x_D(n)\Psi_{\nu,s}(n,t) - \Delta''_{on}\Psi_{\nu,s}(n',t) - t_{\perp}[\delta(n,151) + \delta(n,152) + \delta(n,153) + \delta(n,154)]\Psi_{\nu,s}(n-96,t) - t_{\perp}[\delta(n,155) + \delta(n,156) + \delta(n,157) + \delta(n,158)]\Psi_{\nu,s}(n-62,t)$$

x_D is the position of the sites on the benzene rings under the case of D unit coupling with PS, described as

$$x_D(n) = \begin{cases}(n-96-1)a + u_n & ,151 \le n \le 154 \\ (n-62-1)a + u_n & ,155 \le n \le 158\end{cases}$$

Thirdly, for the case of A unit coupling with PS, the evolution of an electronic state $\Psi_{\nu,s}(n,t)$ depends on the time dependent Schrödinger equation:

For the polymer part,

$$i\hbar\frac{\partial\Psi_{\nu,s}(n,t)}{\partial t} = -t_{n,n+1}\Psi_{\nu,s}(n+1,t) - t_{n-1,n}\Psi_{\nu,s}(n-1,t) + t_3\{\Psi_{\nu,s}(i+3,t)[\delta(\frac{i-(j-1)L-7}{4},int) + \delta(\frac{i-(j-1)L-9}{4},int)] + \Psi_{\nu,s}(i-3,t)\delta(\frac{i-(j-1)L-10}{4},int) + \delta(\frac{i-(j-1)L-12}{4},int)\} + \Delta_{on}\Psi_{\nu,s}(n,t) - \Delta'_{on}\Psi_{\nu,s}(n',t) + eE(t)[(n-1)a + u_n]\Psi_{\nu,s}(n,t) - t_{\perp}[\delta(n,71) + \delta(n,72) + \delta(n,73) + \delta(n,74)]\Psi_{\nu,s}(n+80,t) - t_{\perp}[\delta(n,109) + \delta(n,110) + \delta(n,111) + \delta(n,112)]\Psi_{\nu,s}(n+46,t)$$

And for the PS part,

$$i\hbar\frac{\partial\Psi_{\nu,s}(n,t)}{\partial t} = -t_{n,n+1}\Psi_{\nu,s}(n+1,t) - t_{n-1,n}\Psi_{\nu,s}(n-1,t) + eE(t)x_A(n)\Psi_{\nu,s}(n,t) - \Delta''_{on}\Psi_{\nu,s}(n',t) - t_{\perp}[\delta(n,151)+\delta(n,152)+\delta(n,153) + \delta(n,154)]\Psi_{\nu,s}(n-80,t) - t_{\perp}[\delta(n,155)+\delta(n,156)+\delta(n,157) + \delta(n,158)]\Psi_{\nu,s}(n-46,t)$$

x_A is the position of the sites on the benzene rings under the case of A unit coupling with PS, described as

$$x_A(n) = \begin{cases} (n-80-1)a + u_n & ,151 \le n \le 154 \\ (n-46-1)a + u_n & ,155 \le n \le 158 \end{cases}$$

On the other hand, for the lattice part, the nuclear motion is classically described by the Newtonian equation of motion:

$$M\ddot{u}_n = -K(2u_n - u_{n+1} - u_{n-1}) + 2\alpha[\rho_{n,n+1}(t) - \rho_{n-1,n}(t)] + eE(t)[\rho_{n,n}(t) - 1] - \lambda M\dot{u}_n$$

u_n represents the displacement of the site n, notably, when the n+1th and the n-1th site is on another molecule or inexistent, the items $u_{n+1}(\rho_{n,n+1})$ or $u_{n-1}(\rho_{n-1,n})$ in the Newtonian equation of motion should be 0, because there is no interaction between these sites.

The density matrix $\rho_{n,m}$ is is defined as:

$$\rho_{n,m}(t) = \sum_{\nu,s}\Psi^{*}_{\nu,s}(n,t)\, g_{\nu,s}\sum_{\nu,s}\Psi_{\nu,s}(m,t)$$

Here, $g_{\nu,s}$ is a time-independent distribution function and determined by the initial occupation of the electronic state $|\Psi_{\nu,s}(t)>$. The static electronic eigenequation and the lattice balance equation solved by the Runge-Kutta method of order eight with step-size control.

In addition, a damping term is introduced in Newtonian equation to describe the energy dissipation into the surrounding medium by a tuning a parameter $\lambda = 0.05\ \mathrm{fs}^{-1}$. In all simulations, the values of model parameters are set as $\alpha = 4.1\ \mathrm{eV}\cdot\mathrm{Å}^{-1}$, $K = 21\mathrm{eV}\cdot\mathrm{Å}^{-2}$, $M = 1349.14\mathrm{eV}\cdot\mathrm{fs}^2\cdot\mathrm{Å}^{-2}$, $t_0 = 2.5\mathrm{eV}$, $t_e = 0.05\mathrm{eV}$, $t_1 = 0.05\mathrm{eV}$, $t_2 = 0.05\mathrm{eV}$, $\Delta'_{on} = 0.2\mathrm{eV}$, $\Delta_{on_0} = -0.2\mathrm{eV}$, $\Delta''_{on} = -0.1\mathrm{eV}$, $t_3 = 0.1\mathrm{eV}$.

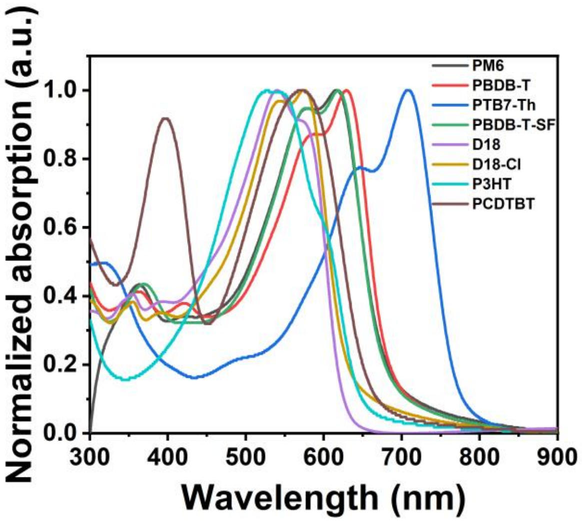

Figure S1. The normalized UV-vis absorption spectra of the donor polymers.

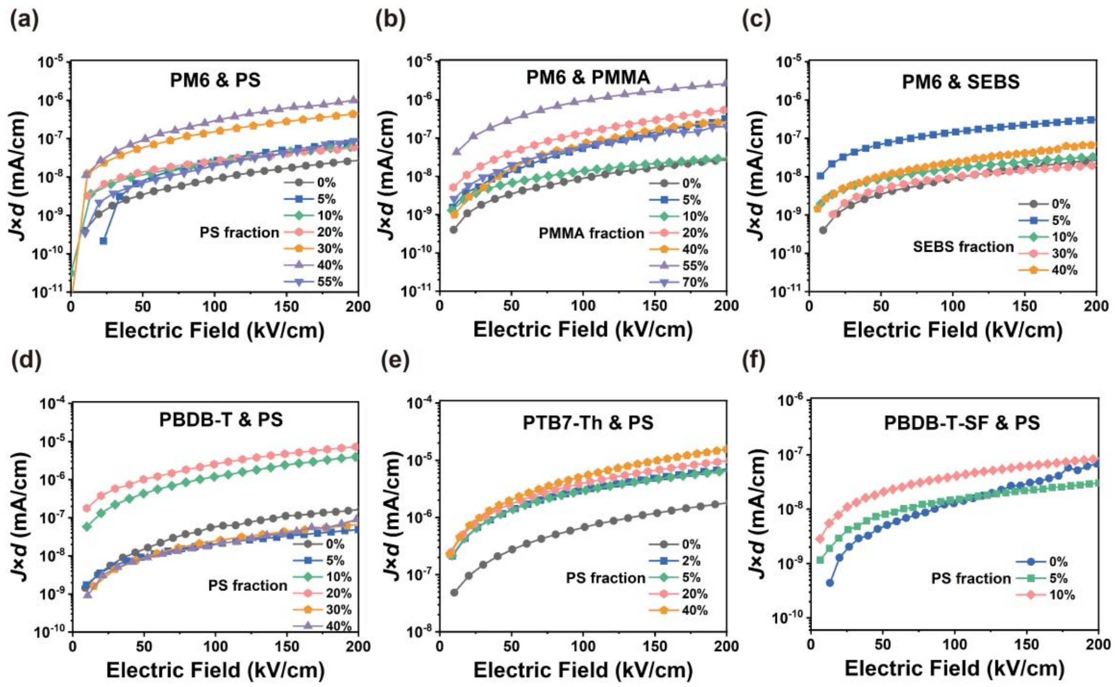

Figure S2. The dependence of $J \times d$ value with the applied electric field for different neat and blending systems using for SCLC analysis. (a) PM6 & PS, (b) PM6 & PMMA, (c) PM6 & SEBS, (d) PBDB-T & PS, (e) PTB7-Th & PS and (f) PBDB-T-SF & PS.

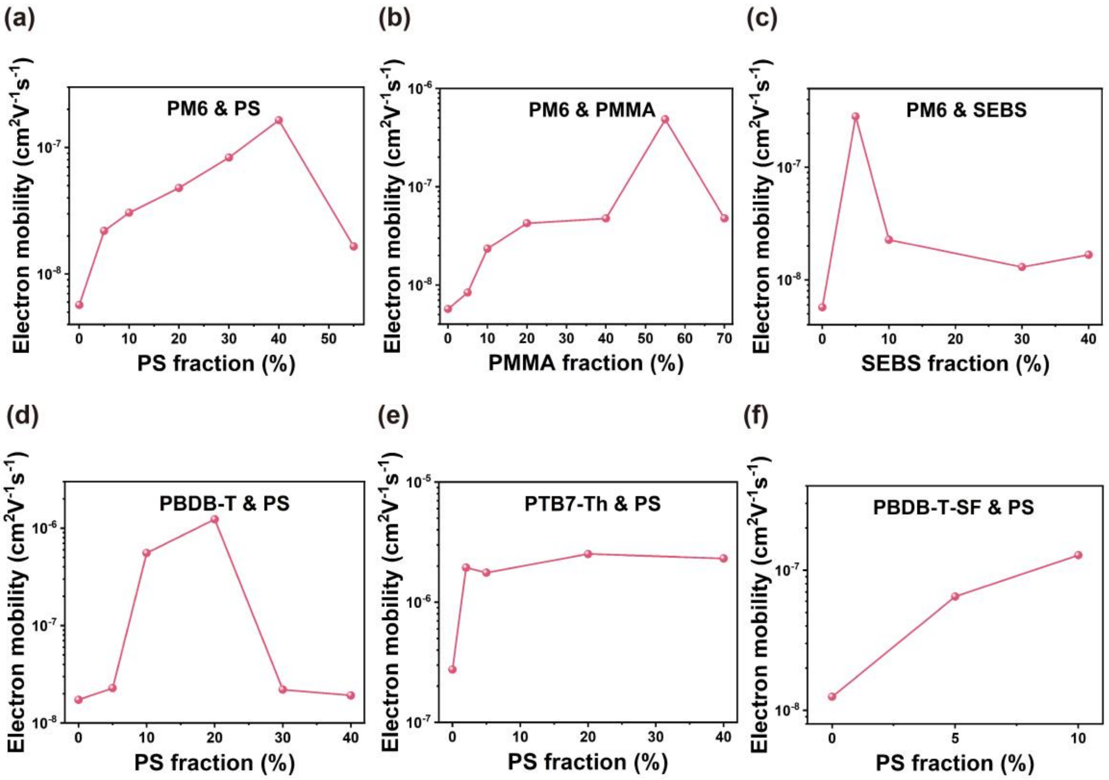

Figure S3. The detailed relationship between electron mobility and insulator fraction in (a) PM6 & PS, (b) PM6 & PMMA, (c) PM6 & SEBS, (d) PBDB-T & PS, (e) PTB7-Th & PS and (f) PBDB-T-SF & PS systems.

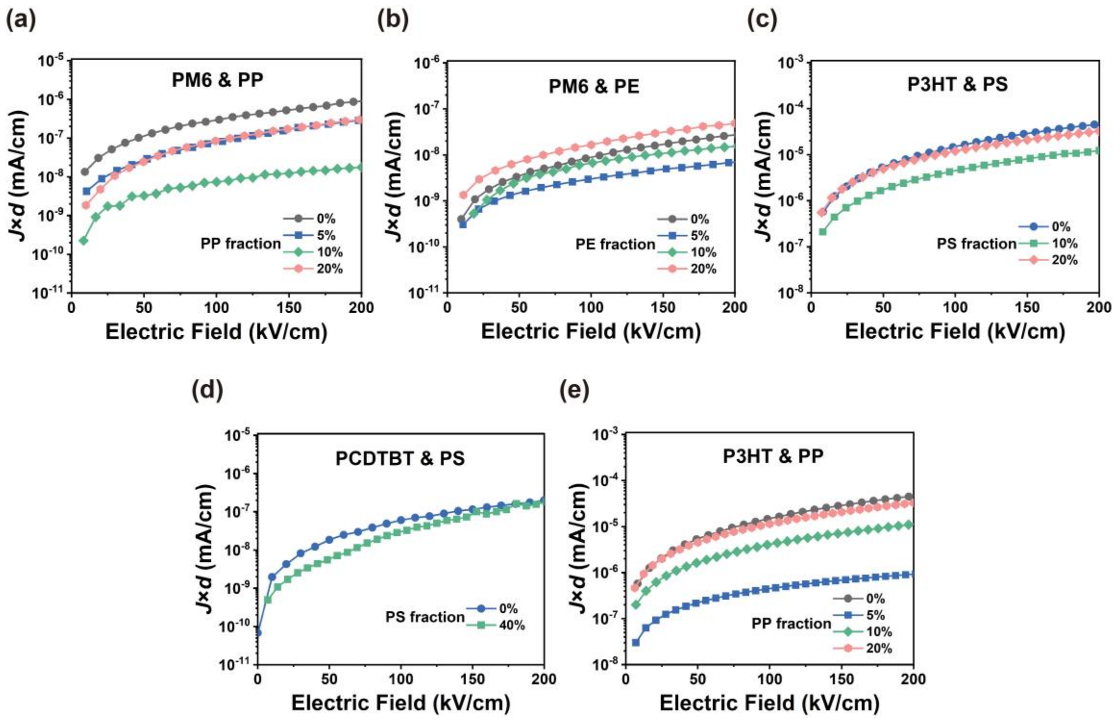

Figure S4. The dependence of $J{\times}d$ value with the applied electric field for different neat and blending systems using for SCLC analysis. (a) PM6 & PP, (b) PM6 & PE, (c) P3HT & PS, (d) PCDTBT & PS and (e) P3HT & PP.

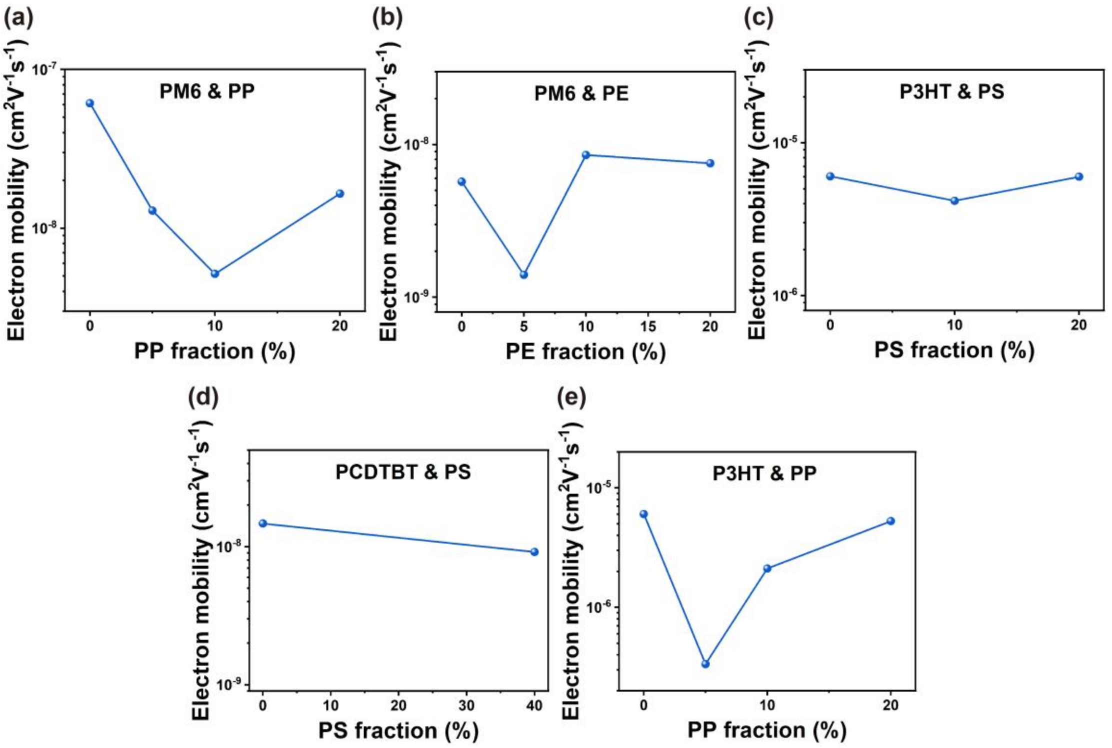

Figure S5. The detailed relationship between electron mobility and insulator fraction in (a) PM6 & PP, (b) PM6 & PE, (c) P3HT & PS, (d) PCDTBT & PS and (e) P3HT & PP systems.

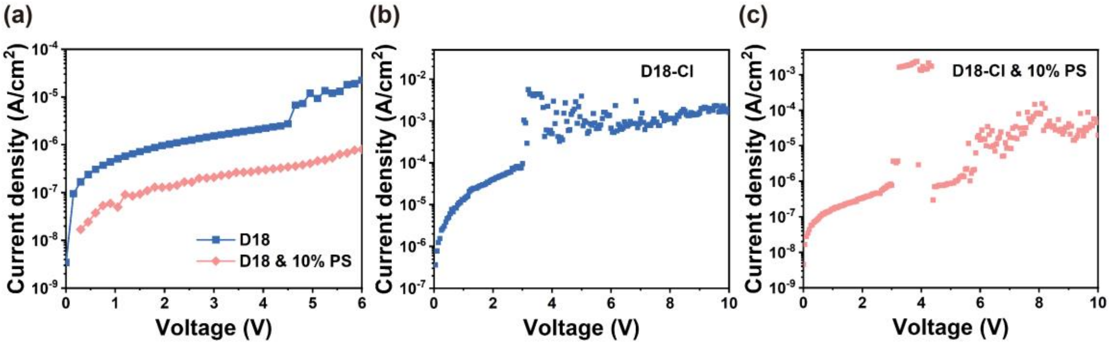

Figure S6. The current density as a function of the applied voltage for (a) D18 and D18 & 10%PS, (b) D18-Cl, (c) D18-Cl & 10%PS systems.

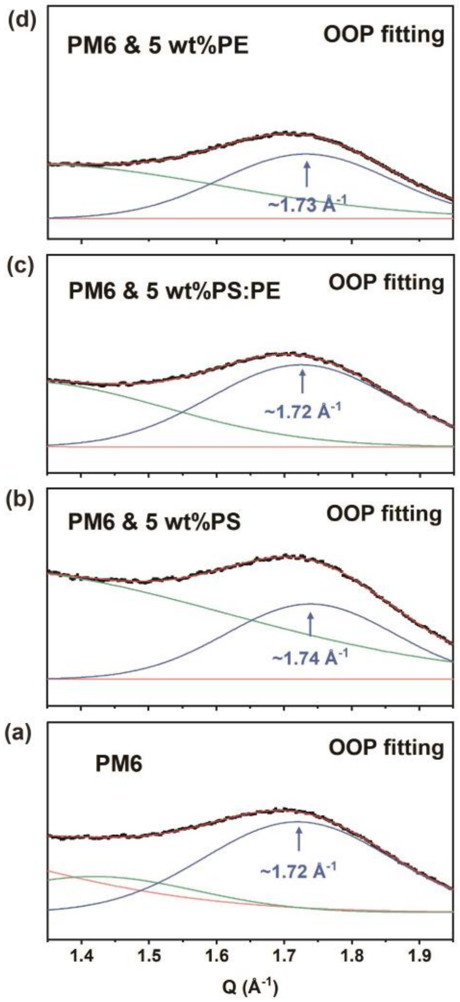

Figure S7. 1D GIWAXS plot and peak splitting results of out-of-plane direction.

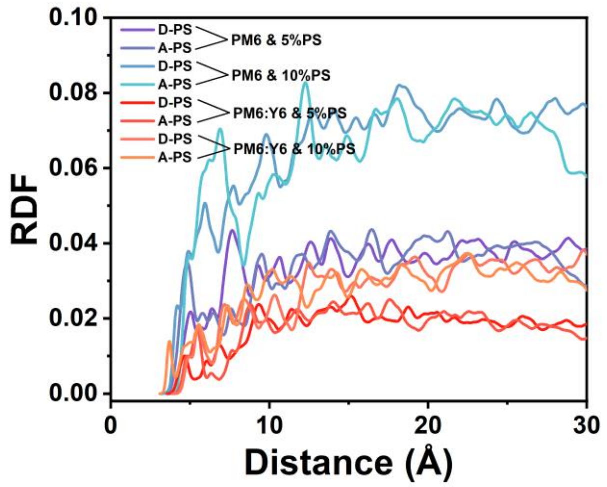

Figure S8. The radial distribution function (RDF) between the D/A unit of PM6 and PS under different systems from MD simulation.

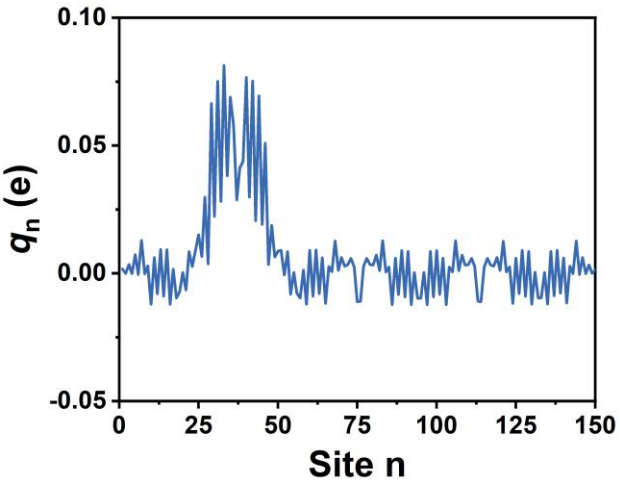

Figure S9. The initial charge density distribution curve of each grid point.

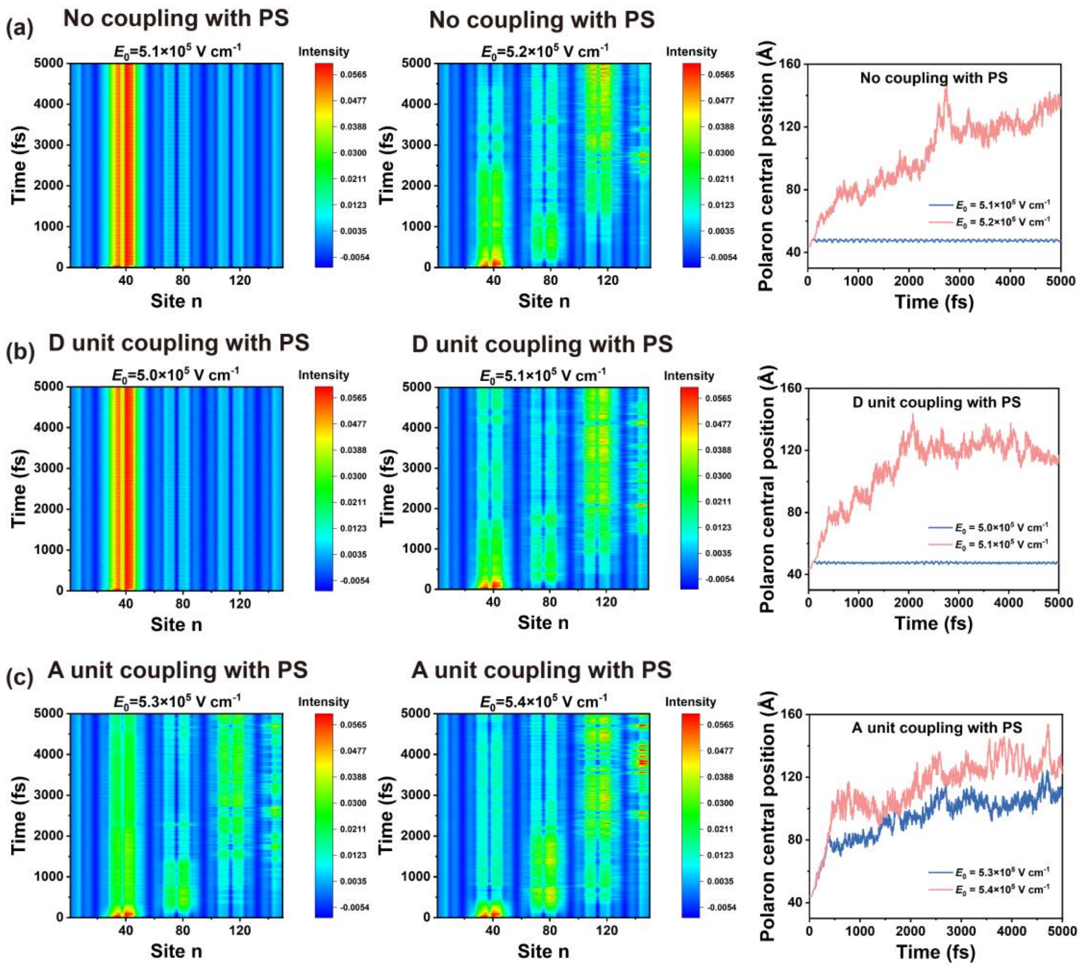

Figure S10. Time evolutions of the net charges on each site and negative polaron center positions of the corresponding molecular models. (a) No coupling with PS, (b) D unit coupling with PS and (c) A unit coupling with PS.

华南国家计量测试中心
广东省计量科学研究院
SOUTH CHINA NATIONAL CENTER OF METROLOGY
GUANGDONG INSTITUTE OF METROLOGY

校 准 结 果
RESULTS OF CALIBRATION

证书编号 NYX202400119 原始记录号 NYX202400119 第 3 页，共 4 页
Certificate No. Record No. Page of

一、外观检查： 符合要求
Apparent Inspecti Pass.

二、测试条件： 温度(25±2)℃；辐照度1000W/m²。
Test conditions: Temperature: (25±2)℃；Irradiance：1000W/m².

三、电流-电压曲线：
The IV curve:

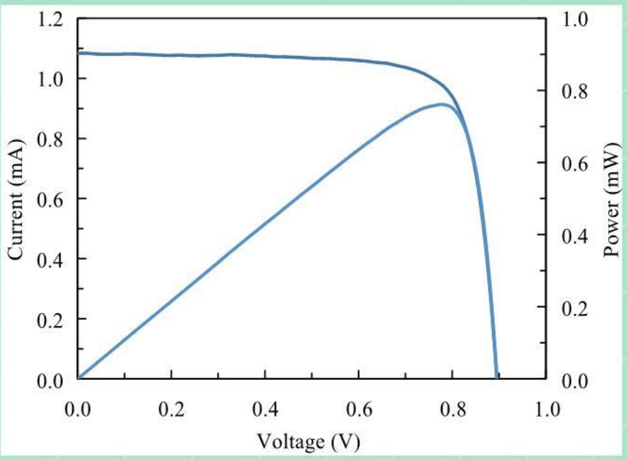

四、光电性能参数：
Results of photoelectric properties:

表1(Table 1)

短路电流密度J_{sc} Short ciruit current density	短路电流I_{sc} Short ciruit current	开路电压V_{oc} Open circuit voltage	填充因子FF Fill factor	最大功率P_m Maximum power	最佳工作电流I_m Optimum working current	最佳工作电压V_m Optimum working voltage	转换效率η Efficiency
mA/cm²	mA	V	%	mW	mA	V	%
27.44	1.084	0.892	78.4	0.758	0.997	0.760	19.18

Figure S11. The certification test report of the (PM6 with 5 wt% PS)/(L8-BO with 3 wt% PS) device at South China National Center of Metrology/Guangdong Institute of Metrology.

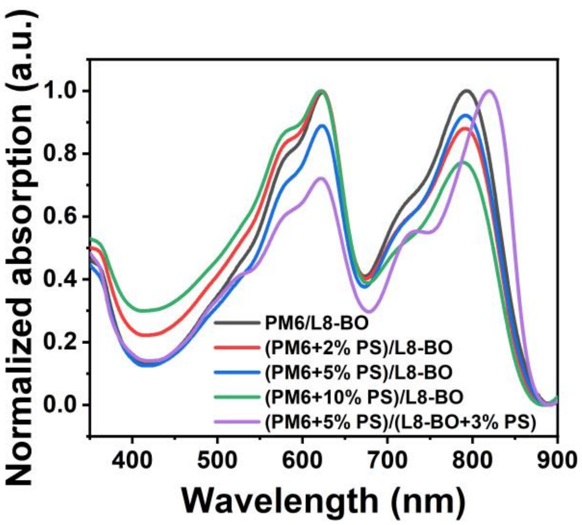

Figure S12. UV-vis absorption images of five different pseudo-bilayer films.

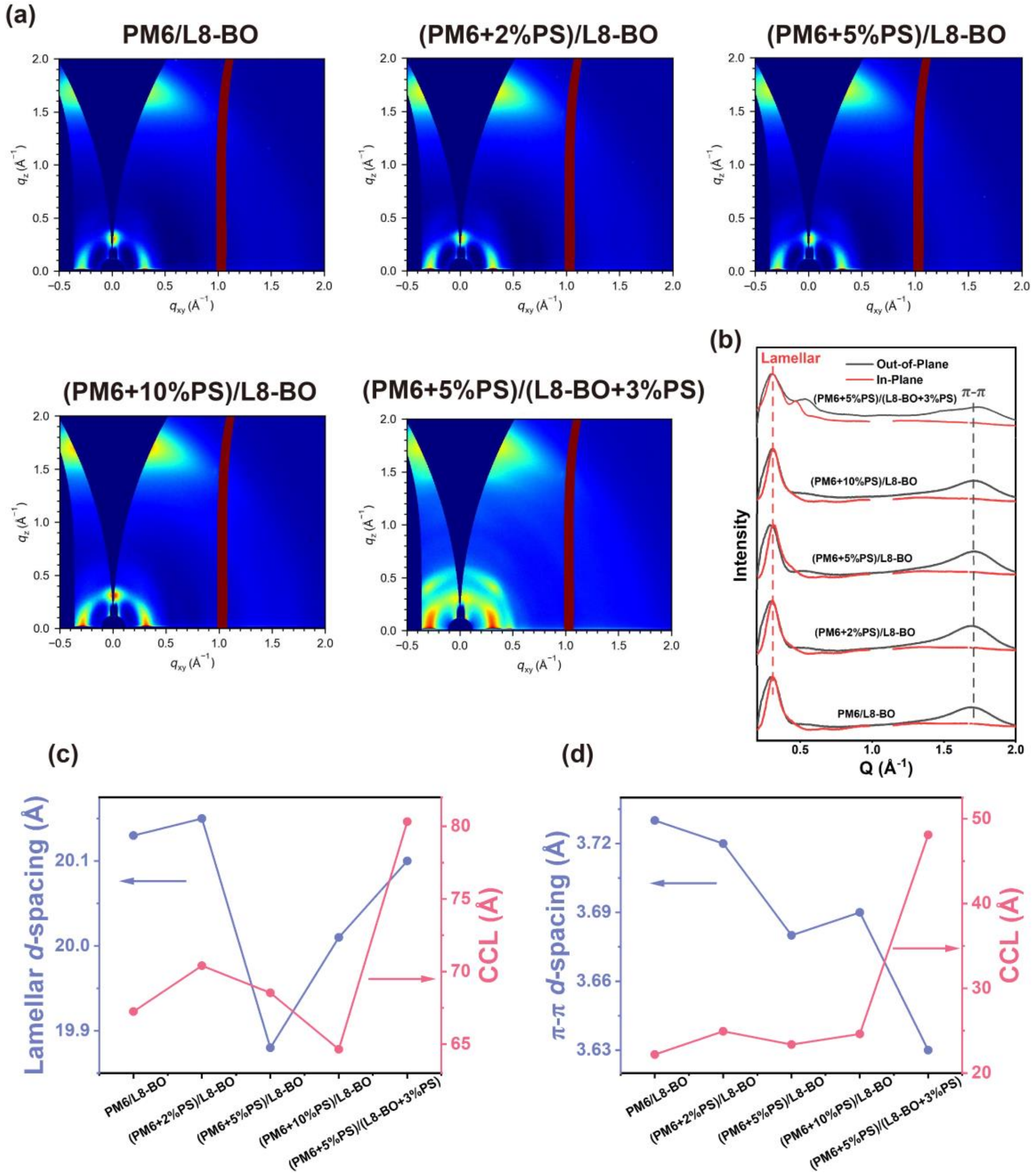

Figure S13. (a) 2D GIWAXS patterns of five different pseudo-bilayer films. (b) Out-of-plane (black) and in-plane (red) linecut profiles of the 2D GIWAXS data. (c), (d) D-spacing (blue symbol) and CCL (red symbol) of (c) lamellar diffraction and (d) π-π peaks for different pseudo-bilayer films.

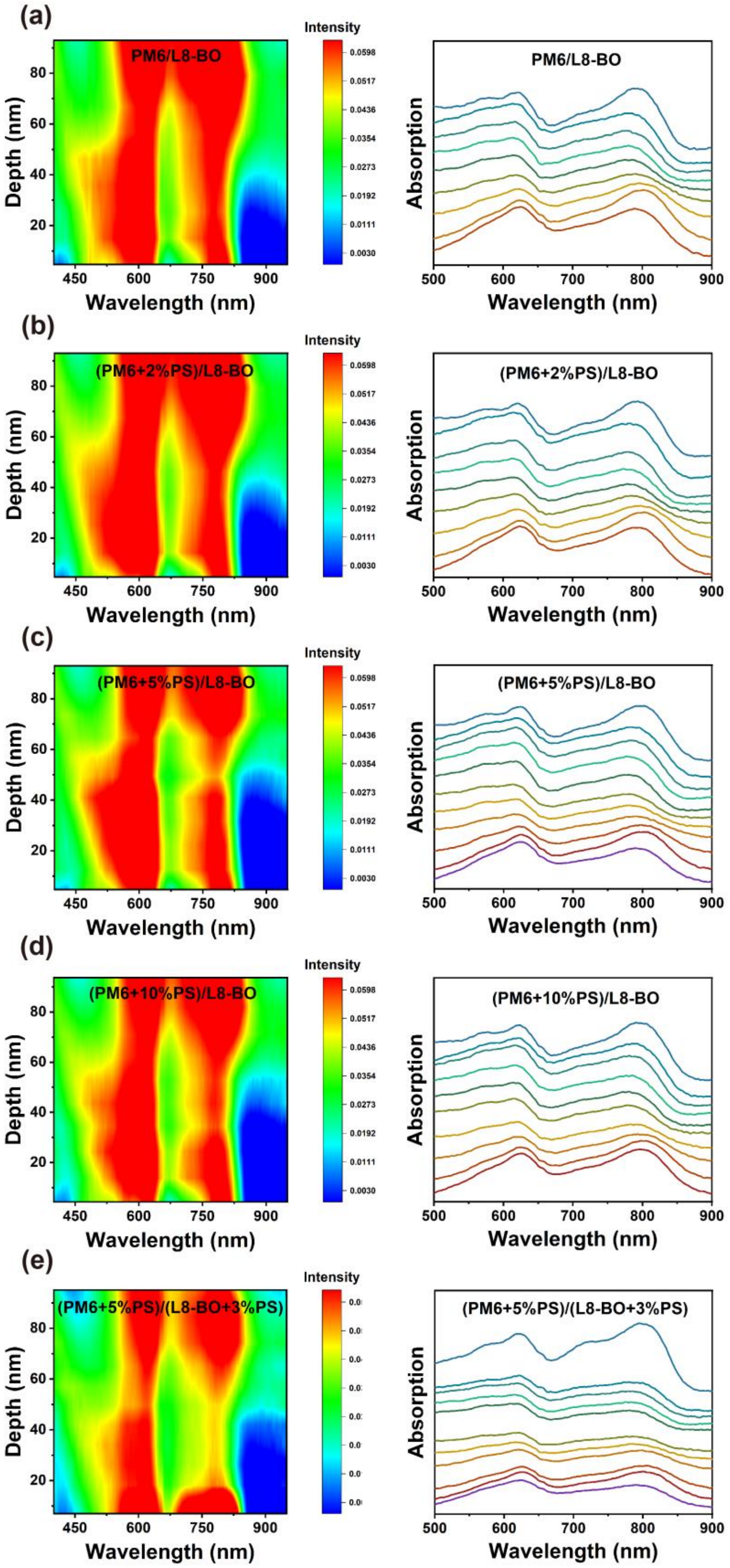
(a)
PM6/L8-BO
Depth (nm)
Wavelength (nm)
Intensity
0.0598
0.0517
0.0436
0.0354
0.0273
0.0192
0.0111
0.0030
Absorption
(b)
(PM6+2%PS)/L8-BO
(c)
(PM6+5%PS)/L8-BO
(d)
(PM6+10%PS)/L8-BO
(e)
(PM6+5%PS)/(L8-BO+3%PS)

Figure S14. Absorption characteristics of the sub-layers for five different pseudo-bilayer films during the FLAS etching process. (a) Two-dimensional, (b) One-dimensional.

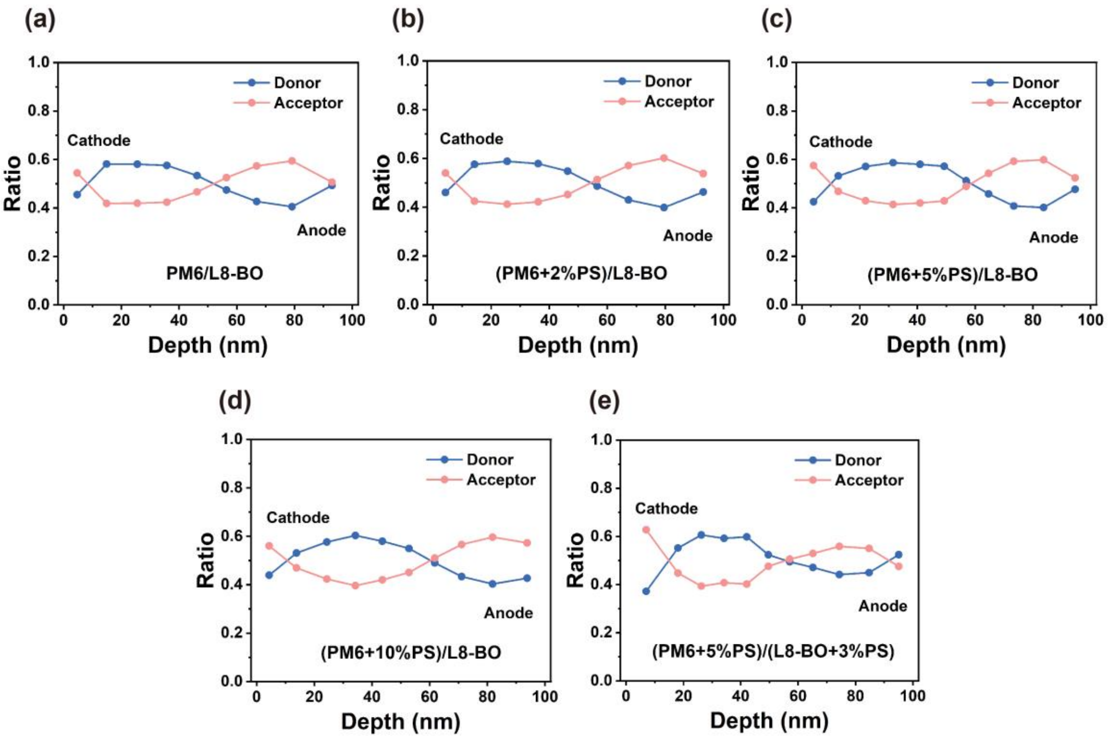

Figure S15. Vertical phase distribution curves of five different pseudo-bilayer films.

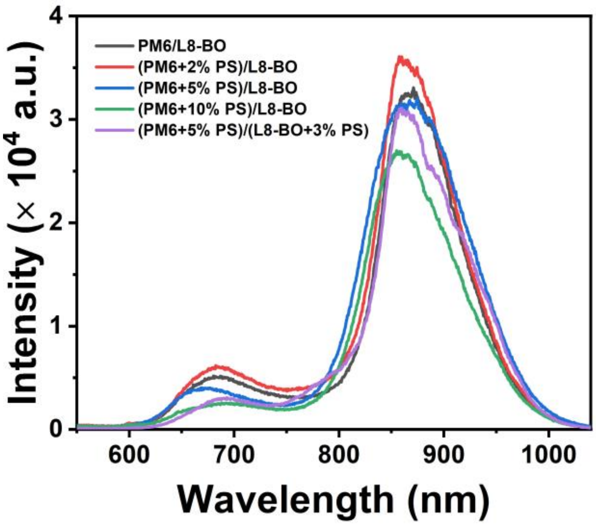

Figure S16. Photoluminescence (PL) images of five different pseudo-bilayer films.

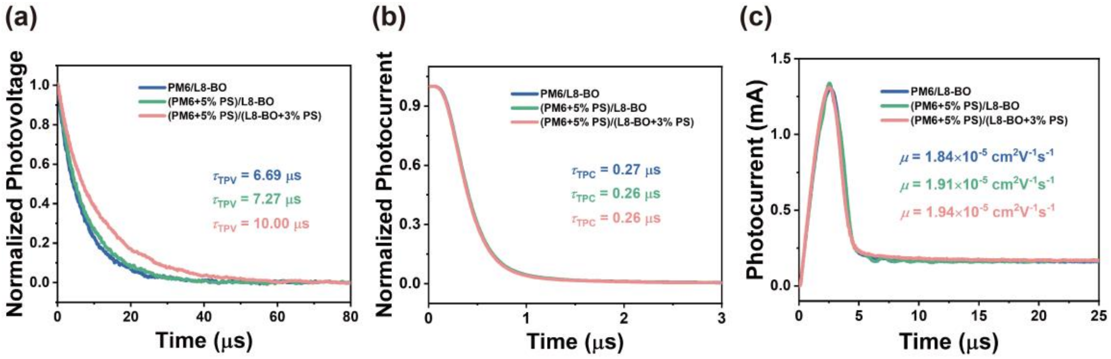

Figure S17. Electrical characteristics of charge carriers. (a) Transient photovoltage; (b) Transient photocurrent; (c) Photo-induced carrier extraction by linearly increasing voltage (photo-CELIV).

Table S1. Summary of GIWAXS π-π stacking information in the OOP direction of neat PM6 and PM6 blending with PS, PS:PE or PE films.

Out-of-Plane				
**System**	$\boldsymbol{q_{(010)}}$ **(Å^{-1})**	***d*-spacing (Å)**	**FWHM (Å^{-1})**	**CCL (Å)**
PM6	1.72	3.65	0.33	17.38
PM6 & 5%PS	1.74	3.61	0.29	19.26
PM6 & 5%PS:PE	1.72	3.64	0.32	17.93
PM6 & 5%PE	1.73	3.63	0.30	18.87

Table S2. Number of close molecular pairs (distance of PS and unit D/A< 6 Å) extracted from MD results.

	**PM6 & 5% PS**	**PM6 & 10% PS**	**PM6:Y6 & 5% PS**	**PM6:Y6 & 10% PS**
D	26	54	7	11
A	29	58	8	17

Table S3. Photovoltaic performance parameters of the pseudo-bilayer devices under AM 1.5G conditions.

**System**	V_{oc} (V)	J_{sc} (mA/cm^2)	J_{cal} (mA/cm^2)	**FF (%)**	**PCE (%)**
(PM6+2%PS)/L8-BO	0.885	27.16	26.45	76.9	18.49 (18.03±0.42)
(PM6+10%PS)/L8-BO	0.889	26.09	25.23	76.8	17.81 (17.58±0.21)

Table S4. Photovoltaic performance parameters of the pseudo-bilayer devices reported in the literatures since 2021.

Devices	PCE (%)	Year	Reference
PM6/N3:$PC_{71}BM$	17.42	2021	7
PM6/BTP-eC9	17.48	2021	8
PNTB6-Cl/N3	17.59	2021	9
PM6/BO-4Cl	17.11	2021	10
PM6/BO-4Cl+25%BTP-S2	18.16		
D18/BS3TSe-4F	18.48	2022	11
D18/BS3TSe-4F:Y6-O	19.03		
D18/L8-BO	19.05	2022	12
PM6/BTP-eC9	17.36	2022	13
PM6+2.5%P-Cl/BTP-eC9	19.10		
PM6/L8-BO	18.72	2023	14
PM6+10%FA-C12/L8-BO	19.02		
D18/L8-BO	18.70	2023	15
D18+0.5%PVK/L8-BO	19.05		
PM6/L8-BO	18.20	2023	16
PM6+0.2%T-2OEH/L8-BO	19.20		
PM6/L8-BO	17.67	2023	17
PM6:BO-4Cl/PM6:L8-BO	19.32		
PM1/L8-BO	18.02	2024	18
PM1/L8-BO+10%PM1	18.81		
PM6/L8-BO	19.03	2024	19
PM6 /L8-BO	18.50	2024	20
PM6+1%L8-BO/L8-BO+1%PM6	19.40		
PM6/BTP-eC9	17.53	2024	21
PM6/BTP-eC9+20%PY-IT	19.41		

Table S5. Summary of Urbach energy (E_u) values obtained from sensitive sub-bandgap external quantum efficiency (s-EQE) for three different pseudo-bilayer films.

System	E_u (meV)
PM6/L8-BO	24.9
(PM6+5%PS)/L8-BO	24.8
(PM6+5%PS)/(L8-BO+3%PS)	24.3

Table S6. Summary of GIWAXS π-π and lamellar stacking information in the OOP and IP direction for different pseudo-bilayer films.

Out-of-Plane

**System**	***q*** **(Å^{-1})**	***d*-spacing** **(Å)**	**FWHM** **(Å^{-1})**	**CCL** **(Å)**
PM6/L8-BO	0.30	21.00	0.17	33.67
	1.69	3.73	0.25	22.20
(PM6+2%PS)/L8-BO	0.30	21.02	0.13	45.08
	1.69	3.72	0.23	24.93
(PM6+5%PS)/L8-BO	0.29	21.34	0.16	36.24
	1.71	3.68	0.24	23.36
(PM6+10%PS)/L8-BO	0.30	20.61	0.11	49.48
	1.70	3.69	0.23	24.61
(PM6+5%PS)/(L8-BO+3%PS)	0.31	20.42	0.10	55.70
	1.73	3.63	0.12	48.10

In-Plane

**System**	***q*** **(Å^{-1})**	***d*-spacing** **(Å)**	**FWHM** **(Å^{-1})**	**CCL** **(Å)**
PM6/L8-BO	0.31	20.13	0.08	67.25
(PM6+2%PS)/L8-BO	0.31	20.15	0.08	70.40
(PM6+5%PS)/L8-BO	0.32	19.88	0.08	68.54
(PM6+10%PS)/L8-BO	0.31	20.01	0.09	64.65
(PM6+5%PS)/(L8-BO+3%PS)	0.31	20.10	0.07	80.32

Table S7. PM6 and L8-BO peak integral intensity ratio exacted from photoluminescence (PL) images.

System	PM6 Intensity Ratio	L8-BO Intensity Ratio
PM6/L8-BO	0.13	0.87
(PM6+2%PS)/L8-BO	0.13	0.87
(PM6+5%PS)/L8-BO	0.09	0.91
(PM6+10%PS)/L8-BO	0.08	0.92
(PM6+5%PS)/(L8-BO+3%PS)	0.07	0.93

References

1. Liu Y, Liu F, Wang H-W *et al.* Sequential deposition: optimization of solvent swelling for high-performance polymer solar cells. *ACS Appl Mater Interfaces* 2014; **7**: 653-61.
2. Chen W, Du Z, Xiao M *et al.* High-performance small molecule/polymer ternary organic solar cells based on a layer-by-layer process. *ACS Appl Mater Interfaces* 2015; **7**: 23190-6.
3. Cheng P, Yan C, Wu Y *et al.* Efficient and stable organic solar cells via a sequential process. *J Mater Chem C* 2016; **4**: 8086-93.
4. Cui Y, Zhang S, Liang N *et al.* Toward efficient polymer solar cells processed by a solution‐processed layer‐by‐layer approach. *Adv Mater* 2018; **30**: 1802499.
5. Sun R, Guo J, Sun C *et al.* A universal layer-by-layer solution-processing approach for efficient non-fullerene organic solar cells. *Energy Environ Sci* 2019; **12**: 384-95.
6. Weng K, Ye L, Zhu L *et al.* Optimized active layer morphology toward efficient and polymer batch insensitive organic solar cells. *Nat Commun* 2020; **11**: 2855.
7. Jiang K, Zhang J, Peng Z *et al.* Pseudo-bilayer architecture enables high-performance organic solar cells with enhanced exciton diffusion length. *Nat Commun* 2021; **12**: 468.
8. Zhang Y, Liu K, Huang J *et al.* Graded bulk-heterojunction enables 17% binary organic solar cells via nonhalogenated open air coating. *Nat Commun* 2021; **12**: 4815.
9. Ning H, Jiang Q, Han P *et al.* Manipulating the solubility properties of polymer donors for high-performance layer-by-layer processed organic solar cells. *Energy Environ Sci* 2021; **14**: 5919-28.
10. Zhan L, Li S, Xia X *et al.* Layer‐by‐layer processed ternary organic photovoltaics with efficiency over 18%. *Adv Mater* 2021; **33**: 2007231.
11. Gao W, Qi F, Peng Z *et al.* Achieving 19% power conversion efficiency in planar‐mixed heterojunction organic solar cells using a pseudosymmetric electron acceptor. *Adv Mater* 2022; **34**: 2202089.

12. Wei Y, Chen Z, Lu G *et al.* Binary organic solar cells breaking 19% via manipulating the vertical component distribution. *Adv Mater* 2022; **34**: 2204718.
13. Zhou M, Liao C, Duan Y *et al.* 19.10% efficiency and 80.5% fill factor layer‐by‐layer organic solar cells realized by 4‐Bis(2‐Thienyl)Pyrrole‐2,5‐Dione based polymer additives for inducing vertical segregation morphology. *Adv Mater* 2022; **35**: 2208279.
14. Ding G, Chen T, Wang M *et al.* Solid additive-assisted layer-by-layer processing for 19% efficiency binary organic solar cells. *Nano-Micro Lett* 2023; **15**: 92.
15. Chen Q, Huang H, Hu D *et al.* Improving the performance of layer‐by‐layer processed organic solar cells via introducing a wide‐bandgap dopant into the upper acceptor layer. *Adv Mater* 2023; **35**: 2211372.
16. Liu C, Fu Y, Zhou J *et al.* Alkoxythiophene‐directed fibrillization of polymer donor for efficient organic solar cells. *Adv Mater* 2023; **36**: 2308608.
17. Wen L, Mao H, Zhang L *et al.* Achieving desired pseudo‐planar heterojunction organic solar cells via binary‐dilution strategy. *Adv Mater* 2023; **36**: 2308159.
18. Tian H, Xu W, Liu Z *et al.* Over 18.8% efficiency of layer‐by‐layer organic photovoltaics enabled by ameliorating exciton utilization in acceptor layer. *Adv Funct Mater* 2024; 2313751.
19. Wu W, Luo Y, Dela Peña TA *et al.* Defining solid additive's pivotal role on morphology regulation in organic solar cells produced by layer‐by‐layer deposition. *Adv Energy Mater* 2024; 2400354.
20. Wang L, Chen C, Fu Y *et al.* Donor–acceptor mutually diluted heterojunctions for layer-by-layer fabrication of high-performance organic solar cells. *Nat Energy* 2024; **9**: 208-18.
21. Zhang Y, Deng W, Petoukhoff CE *et al.* Achieving 19.4% organic solar cell via an in situ formation of p-i-n structure with built-in interpenetrating network. *Joule* 2024; **8**: 509-26.
