# Supplementary material for: Insulator-donor electron wavefunction coupling in pseudo-bilayer organic solar cells achieving a certificated efficiency of 19.18%
Source: Natl Sci Rev. 2024 Oct 30;12(1):nwae385. doi: 10.1093/nsr/nwae385 (PMC11702652; doi:10.1093/nsr/nwae385)
Supplement: nwae385_Supplemental_File [file nwae385_supplemental_file.zip › Teaser Text.docx]

This work offers a novel perspective on the quantum effect of polymeric insulators in organic semiconductors and presents a simple yet effective method for enhancing the performance of organic solar cells.
